# Supplementary material for: Assessing the Accuracy, Completeness and Safety of ChatGPT-4o Responses on Pressure Injuries in Infants: Clinical Applications and Future Implications
Source: Nurs Rep. 2025 Apr 14;15(4):130. doi: 10.3390/nursrep15040130 (PMC12029477; doi:10.3390/nursrep15040130)
Supplement: Supplementary file 1 [file nursrep-15-00130-s001.zip › Table S1. List of 60 questions_tracked.pdf]

| Thematic areas | Questions |
|---|---|
| Definition and Classification | 1. What is meant by a pressure injury in neonates? |
| | 2. What are the main differences between pressure injuries in neonates and adults? |
| | 3. What classifications are used for pressure injuries in neonates? |
| | 4. How are the different stages of pressure injuries identified in a neonate? |
| | 5. Can neonatal pressure injuries be classified based on the depth of the lesion? |
| Risk Factors | 6. What are the main risk factors for the development of pressure injuries in neonates? |
| | 7. How do low birth weight and prematurity affect the risk of pressure injuries? |
| | 8. Is neonatal hypoxia a predisposing factor for pressure injuries? |
| | 9. What clinical conditions make neonates more vulnerable to pressure injuries? |
| | 10. Do medical devices increase the risk of pressure injuries in neonates? |
| Prevention | 11. What preventive strategies can be adopted to reduce pressure injuries in neonates? |
| | 12. How are international guideline recommendations about pressure injuries prevention applied to neonates? |
| | 13. What materials or devices can be used to prevent pressure injuries? |
| | 14. Are there differences in pressure injury prevention strategies between premature and full-term neonates? |
| | 15. What specific protocols about pressure injuries are applied in neonatal intensive care units? |
| Management of Medical Devices | 16. What are the most common medical devices that cause pressure injuries in neonates? |
| | 17. How can the risk of pressure injuries associated with CPAP masks be managed? |
| | 18. What precautions should be taken to prevent pressure injuries from umbilical catheters? |
| | 19. How can pressure injuries related to bandages and casts in neonates be prevented? |
| | 20. How should pressure injuries related to medical devices be documented? |
| Assessment and Monitoring | 21. What are the early signs of a pressure injury in a neonate? |
| | 22. How is the severity of a pressure injury in a neonate assessed? |

| | |
|---|---|
| | 23. What risk assessment tools for pressure injuries are most suitable for neonates? |
| | 24. How frequently should high-risk neonates be monitored for pressure injuries? |
| | 25. Are there standardized protocols for monitoring pressure injuries? |
| Treatment | 26. What treatments are most appropriate for superficial pressure injuries in neonates? |
| | 27. How are advanced-stage pressure injuries managed in neonates? |
| | 28. Is a multidisciplinary approach necessary for the treatment of neonatal pressure injuries? |
| | 29. What dressings are recommended for neonates with fragile skin? |
| | 30. How are advanced dressings for pressure injuries used in neonatal care? |
| Complications | 31. What are the most common complications of neonatal pressure injuries? |
| | 32. Can pressure injuries lead to systemic infections in neonates? |
| | 33. What are the signs of infection in a neonatal pressure injury? |
| | 34. Do pressure injuries affect normal skin development in neonates? |
| | 35. What are the long-term outcomes of neonatal pressure injuries? |
| Role of Nurses | 36. What is the role of the nurse in preventing neonatal pressure injuries? |
| | 37. What specific competencies should a nurse have to manage pressure injuries in neonates? |
| | 38. How should pressure injuries in neonates be documented properly? |
| | 39. What educational tools can be used to train nurses about pressure injuries? |
| | 40. How can parents be involved in the prevention of neonatal pressure injuries? |
| Scientific Evidence | 41. What are the latest scientific findings on the prevention of neonatal pressure injuries? |
| | 42. Are there recent studies on the use of innovative materials for preventing or treating pressure injuries? |
| | 43. What are the most recognized international guidelines on neonatal pressure injuries? |
| | 44. Are there differences between guidelines in different countries on neonatal pressure injuries? |
| | 45. How can the quality of the available scientific evidence about neonatal pressure injuries be assessed? |

| | |
|---|---|
| Legal and Ethical Issues | 46. What are the legal implications of mismanagement of pressure injuries in neonates? |
| | 47. How should adverse events related to pressure injuries in neonates be documented? |
| | 48. What are the ethical responsibilities of nurses in preventing pressure injuries? |
| | 49. How can the balance between skin protection and the clinical management of neonates be achieved? |
| | 50. Are there ethical protocols for research on neonatal pressure injuries? |
| Technological Innovations | 51. What technologies are available to monitor the risk of pressure injuries in neonates? |
| | 52. Can the use of skin sensors prevent pressure injuries in neonates? |
| | 53. Regarding pressure injuries, are there new materials or dressings specifically designed for neonates? |
| | 54. What are the benefits of artificial intelligence in managing neonatal pressure injuries? |
| | 55. How are the cost-benefit analyses of advanced technologies in pressure injury prevention evaluated? |
| Social and Psychological Issues | 56. How can pressure injuries affect the psychological well-being of parents? |
| | 57. Can parents be involved in the management of pressure injuries? |
| | 58. What communication strategies are useful for explaining pressure injuries to parents? |
| | 59. Do pressure injuries affect the bond between parents and neonates? |
| | 60. How can healthcare staff be sensitized to the importance of preventing pressure injuries? |
