# Supplementary material for: Assessing the Accuracy, Completeness and Safety of ChatGPT-4o Responses on Pressure Injuries in Infants: Clinical Applications and Future Implications
Source: Nurs Rep. 2025 Apr 14;15(4):130. doi: 10.3390/nursrep15040130 (PMC12029477; doi:10.3390/nursrep15040130)
Supplement: Supplementary file 1 [file nursrep-15-00130-s001.zip › Table S2. List of ChatGPT-4o-generated responses_tracked.pdf]

1. What is meant by a pressure injury in neonates?

A **pressure injury in neonates** refers to localized damage to the skin and/or underlying tissue, usually over a bony prominence or due to prolonged pressure, friction, shear, or a combination of these factors. Neonates are particularly vulnerable to pressure injuries due to their fragile and underdeveloped skin, immature vascular systems, and limited ability to reposition themselves.

Key Features of Pressure Injuries in Neonates:

1. **Cause:**
   - Prolonged contact with medical devices (e.g., nasal cannulas, CPAP masks, endotracheal tubes).
   - Prolonged immobility or pressure on a specific body area, such as occiput (back of the head), heels, or sacrum.
   - Friction or shear during repositioning or handling.
2. **Risk Factors:**
   - Fragile, thin epidermis with underdeveloped stratum corneum.
   - Reduced subcutaneous fat, which offers less cushioning.
   - Medical conditions (e.g., prematurity, low birth weight, hypoxia).
   - Use of adhesives, medical devices, or poorly padded surfaces.
3. **Common Sites in Neonates:**
   - **Occiput (most common):** Due to prolonged supine positioning.
   - **Nasal bridge or cheeks:** From oxygen masks, CPAP prongs, or endotracheal tubes.
   - **Ears:** From positioning devices or prolonged pressure.
   - **Sacrum, elbows, and heels:** Due to pressure while lying.
4. **Classification (Similar to Adults):** Neonatal pressure injuries are classified using the same stages as adult pressure ulcers:
   - **Stage 1:** Non-blanchable erythema of intact skin.
   - **Stage 2:** Partial-thickness skin loss with exposed dermis.
   - **Stage 3:** Full-thickness skin loss, exposing fat.
   - **Stage 4:** Full-thickness skin and tissue loss with exposed muscle, bone, or tendon.
   - **Unstageable:** Depth obscured by slough or eschar.
   - **Deep Tissue Injury (DTI):** Persistent non-blanchable discoloration.
5. **Prevention:**
   - Frequent repositioning of the neonate.
   - Proper cushioning and padding, especially around bony prominences.
   - Use of non-adhesive or skin-friendly medical devices.
   - Careful skin assessments and early intervention for signs of pressure or irritation.
6. **Management:**
   - Relieve pressure by changing position or adjusting devices.
   - Maintain skin integrity with proper hydration and gentle handling.
   - Use specialized dressings or wound care products as needed.
   - Treat any secondary infections promptly.

By preventing and addressing pressure injuries, healthcare providers can improve neonatal outcomes and reduce complications associated with skin breakdown in this vulnerable population.

2. What are the main differences between pressure injuries in neonates and adults?

Pressure injuries (formerly known as pressure ulcers) in neonates differ from those in adults due to distinct physiological, anatomical, and care-related factors. Here are the main differences:

1. Skin Structure and Vulnerability

- **Neonates**:
  - Their skin is thinner, with an underdeveloped stratum corneum, making it more susceptible to friction, shear, and moisture-related injuries.
  - The dermal-epidermal junction is weaker, increasing the risk of injury from mechanical forces.
- **Adults**:
  - Skin is thicker and more resilient but can thin and lose elasticity with age, especially in older adults.
  - Age-related changes, chronic conditions, and reduced mobility contribute to susceptibility.

2. Risk Factors

- **Neonates**:
  - Prematurity is a significant risk factor as preterm neonates have even less developed skin.
  - Devices such as endotracheal tubes, nasal cannulas, pulse oximeters, and electrodes are common culprits for pressure injuries in neonates.
  - Limited mobility is less of a factor because neonates are usually immobile, but external devices and poor handling play a larger role.
- **Adults**:
  - Pressure injuries are often due to prolonged immobility, poor nutrition, incontinence, and comorbidities (e.g., diabetes, vascular disease).
  - Bed-bound or wheelchair-bound individuals are at higher risk, with bony prominences being common sites (e.g., sacrum, heels).

3. Common Sites

- **Neonates**:
  - Pressure injuries typically occur at sites where medical devices contact the skin, such as the head, nose, and extremities.
  - Occiput is a frequent site due to supine positioning.
- **Adults**:
  - Injuries are more common at bony prominences such as the sacrum, heels, hips, and elbows due to prolonged pressure from lying or sitting.

4. Etiology

- **Neonates**:
  - Medical device-related pressure injuries are predominant.
  - Friction and shear from handling or adhesive removal are critical factors.
- **Adults**:

- Prolonged pressure, shear forces, and moisture (e.g., incontinence-associated dermatitis) are primary causes.

5. Healing and Prognosis

- **Neonates**:
  - Their skin heals faster due to a higher rate of cell turnover, but this is offset by the fragility of their skin and susceptibility to secondary infections.
- **Adults**:
  - Healing can be slower, particularly in older adults or those with chronic illnesses, due to reduced cellular regeneration and impaired circulation.

6. Prevention Strategies

- **Neonates**:
  - Gentle handling, regular repositioning (even minor adjustments), and proper cushioning are crucial.
  - Use of non-adhesive dressings or skin barriers to protect from device-related injuries.
  - Continuous monitoring of devices and their contact points.
- **Adults**:
  - Frequent repositioning and use of pressure-relief mattresses or cushions.
  - Addressing nutritional deficiencies and managing comorbidities.
  - Incontinence care and moisture management.

7. Assessment and Classification

- **Neonates**:
  - Classification of pressure injuries in neonates may be modified, considering their unique physiology. For example, Stage 1 injuries may appear differently due to immature skin tone variations.
- **Adults**:
  - Standard staging (Stages 1–4, unstageable, and deep tissue injury) is typically applied.

In summary, while both populations require careful monitoring to prevent and manage pressure injuries, neonates' unique vulnerabilities necessitate device-focused prevention and gentler care, whereas adults' care focuses more on mobility, pressure redistribution, and systemic health factors.

3. What classifications are used for pressure injuries in neonates?

Pressure injuries in neonates are classified based on the standard **National Pressure Injury Advisory Panel (NPIAP)** staging system, which applies universally but is adapted for neonatal populations due to their unique anatomical and physiological characteristics. The classifications are as follows:

1. Stage 1: Non-blanchable Erythema

- **Characteristics:**
  - Intact skin with localized redness that does not blanch when pressed.
  - In neonates, this can appear as a subtle color change (e.g., redness, pinkness, or even purplish hue in darker skin tones).

2. Stage 2: Partial-Thickness Skin Loss

- **Characteristics:**
  - Partial-thickness loss of skin involving the epidermis and/or dermis.
  - Presents as a shallow, open wound or a serum-filled blister.
  - In neonates, these injuries are often caused by friction or moisture (e.g., from medical devices or adhesives).

3. Stage 3: Full-Thickness Skin Loss

- **Characteristics:**
  - Full-thickness loss of skin exposing subcutaneous tissue.
  - The wound bed may have slough or granulation tissue but no exposed muscle, tendon, or bone.
  - In neonates, this stage can progress rapidly due to their fragile skin and minimal subcutaneous tissue.

4. Stage 4: Full-Thickness Skin and Tissue Loss

- **Characteristics:**
  - Full-thickness loss of skin and tissue with exposed bone, muscle, or tendon.
  - Slough or eschar may be present, and tunneling or undermining can occur.

5. Unstageable Pressure Injury

- **Characteristics:**
  - Full-thickness tissue loss where the wound bed is obscured by slough or eschar.
  - In neonates, eschar may develop more rapidly, particularly if exposed to medical devices.

6. Deep Tissue Pressure Injury

- **Characteristics:**
  - Intact or non-intact skin with persistent discoloration (e.g., purple or maroon) and signs of deep tissue damage.
  - In neonates, this may occur from prolonged pressure or shear over bony prominences.

Specific Considerations for Neonates:

- **Etiology:** Neonatal pressure injuries often result from medical devices (e.g., nasal prongs, endotracheal tubes, CPAP interfaces) rather than immobility.
- **Common Sites:** The head, neck, and areas under medical devices are the most common locations.
- **Skin Immaturity:** Neonates, especially preterm infants, have thinner, more fragile skin that is more prone to injury.

The **Neonatal Skin Risk Assessment Scale (NSRAS)** is often used to identify neonates at risk of pressure injuries, guiding preventive strategies.

4. How are the different stages of pressure injuries identified in a neonate?

Pressure injuries in neonates are classified similarly to those in older children and adults, but with certain considerations due to the unique characteristics of neonatal skin, which is thinner, more fragile, and has less subcutaneous tissue. Identification of the different stages of pressure injuries in neonates follows the National Pressure Injury Advisory Panel (NPIAP) classification system, with adaptations for neonates:

Stage 1: Non-blanchable Erythema

- **Description**: Intact skin with localized redness that does not blanch when pressure is applied.
- **Neonatal Considerations**:
  - Redness may be subtle due to the thin epidermis and immature vasculature.
  - Look for changes in skin color (e.g., pink, red, or purple hues depending on the neonate's baseline skin tone).
  - Skin may feel warmer or cooler than surrounding areas.

Stage 2: Partial-Thickness Skin Loss

- **Description**: Partial-thickness loss of skin involving the epidermis and possibly the dermis. May appear as a shallow open ulcer or a serum-filled blister.
- **Neonatal Considerations**:
  - Blisters can form due to friction from adhesive devices or equipment.
  - Surrounding skin may show signs of maceration or erythema.
  - The injury may appear as a moist, pink wound bed.

Stage 3: Full-Thickness Skin Loss

- **Description**: Full-thickness skin loss involving damage to or loss of subcutaneous tissue. Fat may be visible, but bone, tendon, or muscle are not exposed.
- **Neonatal Considerations**:
  - Subcutaneous fat may be minimal, so the depth of the wound may not be as pronounced.
  - Wounds often develop at pressure points such as the occiput, sacrum, or heels.

Stage 4: Full-Thickness Tissue Loss

- **Description**: Full-thickness loss with exposure of bone, tendon, or muscle.
- **Neonatal Considerations**:
  - Uncommon in neonates due to early intervention; however, if present, such injuries can have significant implications for infection and morbidity.
  - Exposed structures (e.g., bone or tendon) may indicate a severe stage 4 injury.

Unstageable Pressure Injury

- **Description**: Full-thickness tissue loss where the base of the ulcer is obscured by slough or eschar.
- **Neonatal Considerations**:
  - Eschar (dry, dark scabs) may develop more quickly due to the neonate's thin skin and fragile vasculature.
  - Care must be taken to differentiate eschar from normal neonatal skin features, such as desquamation.

Deep Tissue Pressure Injury (DTPI)

- **Description**: Intact or non-intact skin with localized discoloration (e.g., maroon or purple) indicating damage to underlying soft tissue.
- **Neonatal Considerations**:
  - May appear as a dark, purplish discoloration that is easily mistaken for bruising.
  - Commonly associated with medical devices or prolonged pressure on bony prominences.

Common Sites for Neonatal Pressure Injuries:

- **Occiput**: Most common due to lying supine.
- **Nares**: From nasal CPAP or oxygen cannulas.
- **Ears**: Due to pulse oximeter sensors or CPAP straps.
- **Sacrum/Buttocks**: Especially in neonates with prolonged immobility or incontinence.
- **Heels**: Less common, but possible in prolonged supine positioning.

Key Points in Neonatal Pressure Injury Identification:

- Use a gentle, visual, and tactile assessment.
- Consider neonatal-specific risk factors like immobility, medical devices, and fragile skin.
- Early-stage injuries (e.g., Stage 1) require prompt intervention to prevent progression.

Prevention strategies, such as frequent repositioning, using pressure-redistributing surfaces, and minimizing medical device pressure, are critical in neonatal care.

5. Can neonatal pressure injuries be classified based on the depth of the lesion?

Yes, neonatal pressure injuries can be classified based on the depth of the lesion, similar to the classification system used for adults. However, there are some important considerations specific to neonates due to their unique physiology and skin characteristics. The National Pressure Injury Advisory Panel (NPIAP) classification system is often adapted for use in neonates:

1. **Stage 1 (Non-blanchable erythema)**:
   - Intact skin with localized redness that does not blanch when pressed.
   - In neonates, redness may be harder to detect, especially in darker skin tones, and the area may feel warmer or firmer compared to surrounding tissue.
2. **Stage 2 (Partial-thickness skin loss)**:
   - Partial-thickness loss of dermis presenting as a shallow open ulcer or as an intact or ruptured blister.
   - In neonates, this stage may be more fragile and prone to rapid progression if not managed promptly.
3. **Stage 3 (Full-thickness skin loss)**:
   - Full-thickness tissue loss where fat may be visible, but bone, tendon, or muscle is not exposed.
   - Neonates are less likely to have visible fat layers due to their underdeveloped subcutaneous tissue.
4. **Stage 4 (Full-thickness tissue loss)**:
   - Full-thickness tissue loss with exposed bone, tendon, or muscle.
   - Neonates are at high risk of secondary infections at this stage due to their immature immune system.
5. **Unstageable Pressure Injury**:
   - Full-thickness tissue loss where the base of the ulcer is covered by slough or eschar, making depth difficult to determine.
   - Eschar in neonates may appear thinner and softer compared to adults.
6. **Deep Tissue Injury**:
   - Persistent, non-blanchable deep red, maroon, or purple discoloration due to damage to underlying soft tissue.
   - Neonatal skin's thin dermis may make deep tissue injuries less common but potentially more severe.

Considerations for Neonates

- Neonatal skin is thinner, more fragile, and more susceptible to injury due to reduced epidermal-dermal cohesion and underdeveloped barrier function.
- Risk factors include prolonged immobility, medical devices, and poor perfusion.
- Prevention strategies, such as pressure redistribution, careful monitoring of medical devices, and optimizing skin hydration, are critical.

Classification by depth in neonates helps in directing appropriate interventions and preventing progression, but the unique physiology of neonates must always guide assessment and treatment.

6. What are the main risk factors for the development of pressure injuries in neonates?

Pressure injuries in neonates are areas of localized tissue damage caused by prolonged pressure or friction. Neonates, particularly those in intensive care units, are at increased risk due to several physiological and medical factors. The main risk factors include:

1. Prematurity

- Immature skin: Neonates, especially preterm infants, have thin, fragile skin with an underdeveloped stratum corneum, making it more prone to injury.
- Reduced subcutaneous fat: Limited cushioning increases susceptibility to pressure-related damage.

2. Prolonged Immobilization

- Restricted movement due to medical devices, such as ventilators, CPAP, or splints, which can exert continuous pressure on specific areas of the body.
- Inadequate repositioning in incubators or cribs.

3. Medical Devices

- Endotracheal tubes, nasal cannulas, masks, and monitoring equipment can cause device-related pressure injuries (DRPIs), particularly on the nose, face, or extremities.
- Prolonged use of adhesive tapes or electrodes.

4. Poor Perfusion and Oxygenation

- Hypotension, sepsis, or conditions causing poor circulation can lead to tissue ischemia and increase the risk of pressure injuries.
- Hypoxemia reduces tissue resilience to pressure.

5. Nutritional Deficiencies

- Inadequate protein, calorie, and micronutrient intake, common in critically ill neonates, impairs skin integrity and wound healing.

6. Mechanical Ventilation and Oxygen Therapy

- Devices such as nasal CPAP or high-flow oxygen systems exert constant pressure on delicate areas, like the nasal bridge or septum.

7. Prolonged Hospital Stay

- Extended periods in neonatal intensive care units (NICUs) increase exposure to risk factors, including prolonged immobility and use of medical devices.

8. Excessive Moisture

- Skin exposure to urine, stool, or sweat, particularly in diapered infants, causes maceration and increases vulnerability to breakdown.

9. Low Birth Weight

- Very low birth weight (VLBW) infants (<1500 g) have thinner skin and reduced reserves to tolerate pressure or shear forces.

10. Infection and Inflammation

- Systemic or local infections weaken skin integrity and delay healing of minor injuries, increasing the risk of progression to pressure injuries.

11. Shear and Friction

- Frequent handling, repositioning, or poor use of support surfaces can lead to shear forces, especially in neonates with fragile skin.

Prevention Strategies

- Use of pressure-relieving surfaces and repositioning schedules.
- Careful application and regular assessment of medical devices.
- Maintaining skin moisture balance while preventing overhydration.
- Optimizing nutrition and addressing systemic health issues like hypoxia or hypotension.

Regular skin assessments and early interventions are critical to mitigating the risk of pressure injuries in neonates.

7. How do low birth weight and prematurity affect the risk of pressure injuries?

Low birth weight (LBW) and prematurity significantly increase the risk of pressure injuries (PIs) in neonates due to several physiological and clinical factors unique to these populations. Here's a detailed explanation:

1. Underdeveloped Skin Structure

- Premature infants have thinner, more fragile skin compared to term neonates, with incomplete development of the stratum corneum.
- The reduced barrier function makes their skin more susceptible to friction, shear, and breakdown, increasing the risk of pressure injuries.

2. Limited Subcutaneous Fat

- Low birth weight and premature neonates often have minimal subcutaneous fat, which normally cushions bony prominences.
- This makes areas such as the occiput, sacrum, and heels more vulnerable to pressure-induced tissue damage.

3. Reduced Mobility

- Premature infants typically have limited spontaneous movement due to immature neuromuscular development.
- Prolonged immobility in fixed positions increases localized pressure and impairs blood flow, a critical factor in pressure injury development.

4. Impaired Circulation

- The circulatory system of premature infants is immature, with less robust microcirculation, which can lead to ischemia and tissue breakdown under pressure.

5. Medical Interventions

- Premature and low-birth-weight neonates are often subjected to prolonged use of medical devices such as endotracheal tubes, continuous positive airway pressure (CPAP) masks, or intravenous catheters.
- These devices exert localized pressure, significantly contributing to device-related pressure injuries.

6. Increased Dependence on Care

- These infants often require intensive care, including frequent handling, positioning, and invasive procedures. Improper handling or positioning can exacerbate pressure on vulnerable areas.

7. Nutritional Deficits

- Premature neonates may have inadequate nutrition, which delays wound healing and decreases skin resilience, further predisposing them to pressure injuries.

8. Systemic Illnesses

- Premature and LBW infants often have comorbidities such as respiratory distress syndrome, sepsis, or hypotension, which can exacerbate tissue hypoxia and increase PI risk.

9. Impaired Immune Response

- The immature immune system in premature neonates makes them more prone to infections, including those arising from compromised skin integrity.

Prevention Strategies

- **Frequent Repositioning:** Minimizes prolonged pressure on any one area.
- **Skin Protection:** Use of emollients and gentle handling to preserve skin integrity.
- **Device Cushioning:** Appropriate padding and checking device placement regularly.
- **Nutritional Support:** Ensuring adequate protein and calorie intake for skin repair and growth.
- **Risk Assessment:** Regularly assessing for PI risk using neonatal-specific tools like the Braden QD scale.

Understanding these factors and implementing targeted preventive measures are critical to reducing the incidence of pressure injuries in this vulnerable population.

8. Is neonatal hypoxia a predisposing factor for pressure injuries?

Neonatal hypoxia can indeed be considered a predisposing factor for pressure injuries, though its contribution is often indirect and multifactorial. Here's why:

1. **Impaired Tissue Oxygenation**: Hypoxia reduces the oxygen supply to tissues, which can impair cellular metabolism and healing processes. In neonates, especially preterm ones, this vulnerability is heightened due to their immature skin and underlying structures.
2. **Vasoconstriction and Tissue Ischemia**: Neonatal hypoxia often triggers vasoconstriction as a compensatory mechanism to redirect blood flow to vital organs. This can lead to reduced blood flow to peripheral tissues, increasing the risk of ischemic damage in pressure-prone areas.
3. **Compromised Skin Integrity**: Neonates, particularly those with hypoxia, may have underdeveloped or fragile skin. This immaturity makes their skin more susceptible to breakdown under pressure or shear forces.
4. **Prolonged Immobilization**: Hypoxic neonates often require intensive care, which includes prolonged periods of immobilization due to mechanical ventilation or other supportive interventions. Limited movement increases the risk of pressure injuries.
5. **Use of Medical Devices**: Hypoxic neonates are more likely to depend on medical devices (e.g., oxygen masks, tubing, monitors), which can exert localized pressure, leading to device-related pressure injuries.

Preventative Measures:

To mitigate the risk of pressure injuries in neonates with hypoxia:

- **Frequent Repositioning**: Regularly changing the neonate's position to relieve pressure points.
- **Specialized Bedding**: Use pressure-relieving mattresses or pads designed for neonates.
- **Skin Monitoring**: Close and frequent examination of skin, especially around bony prominences and under medical devices.
- **Nutritional Support**: Ensuring optimal nutrition to promote skin integrity and healing.

- **Device Padding**: Applying soft, protective barriers around medical devices to minimize direct pressure on the skin.

Addressing neonatal hypoxia promptly and comprehensively can also reduce its downstream effects, including the risk of pressure injuries.

9. What clinical conditions make neonates more vulnerable to pressure injuries?

Neonates are particularly vulnerable to pressure injuries due to several clinical conditions and unique physiological characteristics. These factors include:

1. Immature Skin and Soft Tissue

- **Thin epidermis**: Neonatal skin is underdeveloped, with a thinner epidermis and weaker dermo-epidermal junction, making it more prone to injury from pressure or friction.
- **Reduced subcutaneous fat**: Neonates, especially preterm infants, have less cushioning to protect against pressure.

2. Immobility

- Neonates are largely immobile and cannot shift positions to relieve pressure, leading to prolonged exposure to pressure points.

3. Medical Devices

- **Device-related pressure injuries (DRPIs)**: Common in neonates due to medical equipment like oxygen masks, nasal cannulae, endotracheal tubes, intravenous catheters, or pulse oximeters.
- The skin under these devices is at higher risk due to constant pressure and moisture accumulation.

4. Critical Illness and Prematurity

- **Prematurity**: Preterm neonates are particularly vulnerable due to underdeveloped skin, inadequate thermoregulation, and lower body reserves.
- **Severe illness**: Critically ill neonates often have impaired circulation, edema, or hypotension, which can compromise skin perfusion and increase susceptibility to pressure injuries.

5. Poor Perfusion

- Neonates with conditions such as sepsis, congenital heart defects, or hypotension may have compromised tissue perfusion, increasing the risk of pressure injuries.

6. Nutritional Deficiencies

- Poor nutritional status, common in preterm and critically ill neonates, impairs skin integrity and healing processes.

7. Moisture-Associated Skin Damage (MASD)

- Prolonged exposure to moisture from urine, feces, or excessive sweating can weaken the skin and predispose neonates to pressure injuries.

8. Use of Adhesives

- Frequent use of adhesive tapes for securing medical devices or dressings can cause skin stripping and increase the risk of pressure-related injuries.

9. Inappropriate Bedding and Positioning

- Hard or poorly cushioned surfaces and improper positioning can lead to focal pressure points, increasing the likelihood of injuries.

10. Infections

- Skin infections or systemic infections such as sepsis can compromise skin integrity and exacerbate pressure injury risk.

Preventive Strategies

To reduce the risk of pressure injuries, focus on:

- **Regular repositioning** to redistribute pressure.
- **Use of pressure-relieving devices**, such as gel pads or specialized neonatal mattresses.
- **Monitoring for early signs of injury** at high-risk sites.
- **Skin barrier products** to reduce MASD.
- Careful placement and monitoring of medical devices.

By addressing these risk factors, pressure injuries in neonates can often be prevented or minimized.

10. Do medical devices increase the risk of pressure injuries in neonates?

Yes, medical devices can increase the risk of pressure injuries in neonates. Neonates are particularly vulnerable to pressure injuries due to their fragile skin, immature physiological systems, and limited ability to reposition themselves. Several factors associated with medical devices contribute to this increased risk:

1. Fragile Neonatal Skin

- Neonatal skin is thinner, has less subcutaneous fat, and is more prone to tearing and injury.
- Prolonged contact or friction with medical devices can lead to skin breakdown.

2. Immobility

- Neonates are often unable to reposition themselves, leading to sustained pressure on areas in contact with medical devices.

3. Medical Device Features

- Devices such as nasal cannulas, endotracheal tubes, CPAP masks, IV catheters, and monitoring electrodes can exert localized pressure.
- Poorly fitted or rigid devices may cause excessive pressure or shear forces, increasing the risk of injury.

4. Moisture Accumulation

- Devices that trap moisture (e.g., under adhesive tapes or masks) can lead to maceration and weaken the skin barrier.

5. Prolonged Use

- Extended use of medical devices without proper monitoring and repositioning increases the likelihood of pressure injuries.

Common Sites of Device-Related Pressure Injuries

- **Face and head:** From CPAP masks, nasal prongs, and hats.
- **Extremities:** From IV lines and splints.
- **Back and buttocks:** From sensors, probes, or electrodes.

Preventative Strategies

1. **Regular Skin Assessments:**
    - Frequent monitoring for signs of redness, swelling, or breakdown.
2. **Proper Fitting and Positioning of Devices:**
    - Ensure devices are correctly sized and not overly tight.
3. **Pressure-Relief Interventions:**
    - Use gel pads, foam dressings, or silicone barriers under devices.
4. **Frequent Repositioning:**
    - Adjust device placement periodically to relieve pressure.
5. **Skin Protection:**
    - Apply protective barriers or dressings to areas in contact with devices.
6. **Moisture Management:**
    - Keep the skin dry and clean, particularly under adhesive dressings or close-fitting devices.

By identifying high-risk neonates and implementing preventative measures, healthcare providers can significantly reduce the incidence of device-related pressure injuries in this vulnerable population.

**11. What preventive strategies can be adopted to reduce pressure injuries in neonates?**
Preventing pressure injuries in neonates is crucial due to their delicate skin, limited mobility, and increased susceptibility to injury. Effective preventive strategies focus on risk assessment, skin protection, and minimizing pressure and friction. Here are some key approaches:<br><br>1. Risk Assessment<br><br>Identify High-Risk Neonates: Use validated neonatal risk assessment tools (e.g., Neonatal Skin Risk Assessment Scale) to identify neonates at higher risk.<br><br>Monitor Continuously: Regularly reassess the neonate's condition, especially during changes in care or health status.<br><br>2. Skin Protection<br><br>Moisturize Skin: Use gentle, fragrance-free moisturizers to prevent dryness and enhance skin integrity.<br><br>Barrier Protection: Apply barrier creams or films to protect areas prone to moisture-associated skin damage, such as the perianal region.<br><br>Gentle Cleansing: Cleanse skin with pH-balanced, non-irritating cleansers and pat dry rather than rubbing.<br><br>3. Pressure Relief<br><br>Frequent Repositioning: Turn neonates at least every 2-4 hours to redistribute pressure. Use a schedule to ensure consistency.<br><br>Support Surfaces: Use pressure-relieving mattresses or overlays designed for neonates to minimize pressure points.<br><br>Positioning Aids: Utilize soft, padded supports and gel pillows to offload pressure from bony prominences, such as the occiput.<br><br>4. Minimizing Medical Device-Related Pressure Injuries<br><br>Proper Padding: Place soft padding under medical devices like nasal cannulas, feeding tubes, and IV lines.<br><br>Secure but Loose Fittings: Ensure medical devices are secure but not overtight, which can cause pressure and friction.<br><br>Regular Device Checks: Frequently assess skin under and around devices for early signs of injury and reposition devices if feasible.<br><br>5. Education and Training

Staff Education: Train caregivers and healthcare staff on neonatal skin care and pressure injury prevention.

Parental Involvement: Educate parents on gentle handling and recognizing early signs of skin breakdown.

6. Nutrition and Hydration

Adequate Nutrition: Ensure neonates receive appropriate nutrition to support skin integrity and wound healing.

Hydration: Maintain adequate hydration to prevent skin dryness and cracking.

7. Monitoring and Documentation

Early Detection: Regularly inspect the skin for redness, blanching, or other early signs of pressure injury.

Documentation: Maintain detailed records of skin assessments and preventive measures taken.

8. Environmental Control

Temperature Regulation: Avoid excessive heat or cold, which can compromise skin integrity.

Avoid Prolonged Contact with Moisture: Manage incontinence and excessive sweating to prevent maceration.

9. Minimizing Shear and Friction

Careful Handling: Use gentle techniques during repositioning to avoid dragging the neonate's skin against surfaces.

Appropriate Linen Use: Ensure bedding is smooth and free of wrinkles to reduce friction.

10. Multidisciplinary Approach

Involve a multidisciplinary team, including neonatologists, nurses, dietitians, and wound care specialists, to implement and monitor preventive strategies effectively.

Proactive implementation of these strategies can significantly reduce the incidence of pressure injuries in neonates, ensuring better outcomes and reducing morbidity.

12. How are international guideline recommendations about pressure injuries prevention applied to neonates?

International guidelines for pressure injury prevention generally target adult and pediatric populations, but their principles can be adapted for neonates, with modifications accounting for the unique anatomical, physiological, and developmental characteristics of this population. Here's how these recommendations are applied to neonates:

1. Risk Assessment

- **Guidelines** recommend systematic risk assessment using validated tools.
- **Application to Neonates**:
  - Tools like the Neonatal Skin Risk Assessment Scale (NSRAS) or Braden Q Scale (modified for neonates) can be used.
  - Risk factors in neonates include prematurity, low birth weight, prolonged immobility, medical devices, and critical illness.

2. Skin Protection

- **Guidelines** emphasize maintaining skin integrity.
- **Application to Neonates**:
  - Use pH-balanced cleansers and avoid harsh soaps.
  - Minimize the use of adhesives; use hydrocolloid or silicone-based dressings to secure devices.
  - Avoid excessive bathing, as neonates' skin is more fragile and prone to damage.

3. Repositioning and Pressure Redistribution

- **Guidelines** stress the importance of frequent repositioning to alleviate pressure.
- **Application to Neonates**:
  - Reposition neonates at least every 2-4 hours.
  - Use specialized neonatal mattresses or gel pads to redistribute pressure.
  - Avoid direct pressure from medical devices (e.g., oxygen tubing, nasogastric tubes).

4. Nutritional Support

- **Guidelines** highlight the role of adequate nutrition in skin health.
- **Application to Neonates**:
  - Ensure optimal nutrition, including adequate protein and caloric intake, to promote growth and repair.
  - For premature or critically ill neonates, coordinate with a neonatal nutritionist.

5. Medical Device-Related Pressure Injuries (MDRPIs)

- **Guidelines** recognize the risk of MDRPIs.
- **Application to Neonates**:
  - Regularly assess and reposition devices (e.g., endotracheal tubes, CPAP interfaces).
  - Use protective barriers like foam or silicone cushions under devices.

6. Education and Multidisciplinary Approach

- **Guidelines** recommend training healthcare staff on pressure injury prevention.
- **Application to Neonates**:

- Train neonatal healthcare teams on skin care, risk assessment, and early intervention.
- Engage parents in care routines, such as monitoring skin integrity during diaper changes.

7. Monitoring and Early Intervention

- **Guidelines** advise regular skin assessments to detect early signs of pressure injury.
- **Application to Neonates**:
  - Conduct skin checks during routine care.
  - Document skin condition using standardized protocols and escalate concerns promptly.

8. Individualized Care

- Neonates require a tailored approach due to their vulnerability.
  - Account for gestational age and specific comorbidities when implementing guidelines.
  - Monitor for potential side effects of preventive measures (e.g., skin breakdown from adhesives).

By adapting these principles to the neonatal context, healthcare providers can effectively prevent pressure injuries in this high-risk population. Multidisciplinary collaboration and regular updates to protocols based on emerging evidence are essential for success.

13. What materials or devices can be used to prevent pressure injuries?

Preventing pressure injuries involves using various materials and devices that reduce pressure on vulnerable areas, improve support, and promote skin health. Here's a detailed list:

1. Support Surfaces

- **Specialized Mattresses and Overlays**
  - Foam mattresses (high-density or memory foam).
  - Gel-filled mattresses or overlays.
  - Air-fluidized beds.
  - Low-air-loss mattresses.
  - Alternating-pressure mattresses or overlays.
- **Cushions**
  - Gel, foam, or air cushions for wheelchairs or chairs.

2. Protective Padding

- **Heel Protectors**
  - Foam heel protectors.
  - Gel heel pads.
  - Suspension boots (keep heels off the bed surface).
- **Elbow Protectors**

- Foam or padded sleeves.

3. Positioning Devices

- **Wedges and Rolls**
  - Foam wedges to maintain side-lying positions.
  - Cylindrical rolls for limb support and to prevent contact between knees or ankles.
- **Turning and Repositioning Systems**
  - Lateral turning devices.
  - Sliding sheets or repositioning aids for easier handling.

4. Dressings

- **Prophylactic Dressings**
  - Silicone foam dressings (commonly applied to high-risk areas like the sacrum and heels).

5. Skin Care Products

- **Moisture Barriers**
  - Zinc oxide or petroleum-based creams.
  - Liquid skin protectants.
- **Moisturizers**
  - Hydrating lotions to prevent dryness and cracking.
- **Cleanser Alternatives**
  - pH-balanced cleansers instead of soap to reduce irritation.

6. Nutrition Support

- **Pressure Injury Prevention Supplements**
  - Protein-rich dietary supplements or high-calorie drinks.
  - Zinc and vitamin C supplements to support skin integrity.

7. Monitoring Devices

- **Pressure Sensors**
  - Wearable or bed-integrated devices to alert caregivers to prolonged pressure areas.

8. Other Support Tools

- **Shear-Reducing Devices**
  - Reclining or tilt-in-space chairs.
  - Wheelchair cushions with anti-shear designs.
- **Lifting Aids**
  - Transfer boards or hoists to reduce friction and shear during repositioning.

Key Considerations

- Regular repositioning (every 2 hours for bed-bound patients).
- Inspecting skin daily for early signs of pressure damage.
- Individualized device selection based on patient needs, mobility, and risk level.

Combining these materials and devices with routine care and monitoring significantly reduces the risk of pressure injuries.

14. Are there differences in pressure injury prevention strategies between premature and full-term neonates?

Yes, there are differences in pressure injury prevention strategies between premature and full-term neonates due to the unique vulnerabilities of premature neonates. These differences stem from their physiological immaturity, underdeveloped skin, and higher risk of complications. Below are some key distinctions:

1. Skin Fragility

- **Premature Neonates**: Have thinner, less keratinized skin with an underdeveloped stratum corneum, making them highly susceptible to injury. Preventive strategies include:
  - Minimal adhesive use (or use of hydrocolloid-based adhesives).
  - Application of protective barriers (e.g., silicone dressings) over bony prominences.
  - Avoidance of frequent repositioning unless medically necessary.
- **Full-Term Neonates**: Their skin is more developed, providing greater resilience. However, gentle handling and appropriate padding remain essential.

2. Susceptibility to Pressure and Shear Forces

- **Premature Neonates**: Due to reduced subcutaneous fat and immature vasculature, they are highly prone to pressure and shear injuries.
  - Use of pressure-redistributing surfaces, such as gel pads or specialty mattresses.
  - Frequent but careful repositioning (every 2–4 hours), ensuring minimal shear during movement.
- **Full-Term Neonates**: While less prone to shear injuries, repositioning and pressure redistribution are still necessary to prevent injuries, particularly in cases requiring prolonged monitoring or treatment.

3. Medical Device-Related Pressure Injuries (MDRPIs)

- **Premature Neonates**: Devices like CPAP masks, endotracheal tubes, or IV lines are commonly used, increasing MDRPI risk.
  - Vigilant monitoring of device placement and securement without over-tightening.
  - Regular assessment of pressure points around devices.
- **Full-Term Neonates**: At risk primarily in cases requiring medical devices. Monitoring and adjustments are also critical but may be less frequent than in premature neonates.

4. Thermoregulation and Moisture Management

- **Premature Neonates**: Their impaired thermoregulation increases the need for humidity-controlled environments, which can exacerbate moisture-associated skin damage.
  - Use of breathable barriers and careful moisture control.
  - Avoiding excess humidity near pressure points.
- **Full-Term Neonates**: Generally better at thermoregulation, but moisture management remains important, especially in diaper areas.

5. Parental Education and Involvement

- **Premature Neonates**: Parents may need more specialized education due to the fragility of the neonate and the complexity of care in NICU settings.
- **Full-Term Neonates**: Education focuses on general skin care and awareness of pressure injury risks during hospital stays.

6. Nutrition and Healing Support

- **Premature Neonates**: Often require specialized nutritional support to promote skin integrity and healing (e.g., fortified human milk or parenteral nutrition).
- **Full-Term Neonates**: Typically have more robust nutritional reserves but still require adequate feeding to support recovery.

By tailoring prevention strategies to the specific needs of premature and full-term neonates, healthcare providers can significantly reduce the risk of pressure injuries in these vulnerable populations.

15. What specific protocols about pressure injuries are applied in neonatal intensive care units?

In neonatal intensive care units (NICUs), pressure injury prevention protocols are tailored to the unique physiology and vulnerabilities of neonates, especially premature and low-birth-weight infants. Here are specific protocols typically applied:

1. Risk Assessment

- Use of validated tools like the **Braden Q Scale** or **Neonatal Skin Risk Assessment Scale (NSRAS)** to identify neonates at risk of pressure injuries.
- Regular reassessments as clinical conditions change.

2. Skin Integrity Monitoring

- **Daily skin checks** for early signs of pressure injury, such as redness or blanching.
- Documentation of skin condition in nursing records during each shift.

3. Positioning and Repositioning

- Frequent repositioning, typically every 2–3 hours, to alleviate pressure on specific areas.
- Use of positioners or rolls to maintain appropriate posture and reduce pressure on bony prominences (e.g., occiput, sacrum).
- Avoidance of prolonged positioning on medical devices (e.g., CPAP masks, ventilator tubes).

4. Support Surfaces

- Use of pressure-relieving mattresses, overlays, or gel pads to reduce friction and shear.
- Incorporation of fluidized or air-filled mattresses for extremely high-risk infants.

5. Device Management

- Proper fitting and padding of medical devices such as endotracheal tubes, CPAP interfaces, or pulse oximeters to minimize pressure points.
- Frequent repositioning or adjustments of devices to redistribute pressure.

6. Moisture Management

- Prompt management of **incontinence, perspiration, or wound exudate** to prevent moisture-associated skin damage.
- Application of barrier creams or films to protect skin from excessive moisture.

7. Nutritional Support

- Ensuring adequate nutrition, as poor nutritional status can impair skin integrity and wound healing.
- Monitoring serum protein levels (e.g., albumin) in very premature infants.

8. Use of Skin Barriers and Protective Dressings

- Application of silicone-based foam dressings on high-risk areas (e.g., occiput, heels) to redistribute pressure and reduce shear forces.
- Transparent films or hydrocolloid dressings to protect from friction without excessive adhesion.

9. Education and Training

- Comprehensive training for NICU staff on skin integrity protocols, device-related injury prevention, and early recognition of pressure injuries.
- Parental education to involve families in positioning and care routines when appropriate.

10. Interdisciplinary Approach

- Regular consultation with wound care specialists or dermatologists for complex cases.

- Collaboration with dietitians, physical therapists, and occupational therapists for holistic care.

11. Documentation and Reporting

- Consistent documentation of all pressure injuries, including staging, location, and interventions taken.
- Root cause analysis for pressure injuries to improve protocols and reduce recurrence.

By implementing these protocols, NICUs strive to minimize the incidence of pressure injuries while promoting optimal care for neonates with fragile skin.

16. What are the most common medical devices that cause pressure injuries in neonates?

Medical devices are a common cause of pressure injuries in neonates, primarily because their skin is fragile and their subcutaneous tissue is underdeveloped. The most common medical devices associated with pressure injuries in neonates include:

1. **Nasal Continuous Positive Airway Pressure (nCPAP) Prongs and Masks**
    - Frequently used for respiratory support, these devices can cause pressure injuries to the nasal bridge, nares, and septum due to prolonged contact and pressure.
2. **Endotracheal Tubes and Securing Devices**
    - Used for mechanical ventilation, they can cause injuries around the mouth, lips, and cheeks from prolonged contact or tightly secured fixations.
3. **Pulse Oximeter Probes**
    - Continuous monitoring with these probes can lead to injuries on the foot, hand, or digit if left in one position for too long.
4. **Intravenous (IV) Catheters and Peripheral Intravenous Lines (PIVs)**
    - Devices like PIVs, when secured tightly or improperly, may lead to pressure injuries at the insertion site or under the adhesive dressing.
5. **Gastrostomy Tubes (G-tubes) and Nasogastric (NG) Tubes**
    - These tubes may cause injuries around the insertion site, especially when the skin underneath is not protected or the device is improperly secured.
6. **Electrode Pads and ECG Leads**
    - Prolonged contact or improper placement of adhesive electrodes can result in injuries, particularly on fragile or premature neonate skin.
7. **Splints and Orthotic Devices**
    - Devices used to stabilize limbs or joints can create pressure points, particularly if padding is inadequate or the device is too tight.
8. **Headgear for Ventilation or Monitoring**
    - Hats or straps used to secure CPAP masks or monitoring devices can cause pressure injuries on the scalp or forehead.
9. **Orthopedic Devices (e.g., Pavlik Harness, Plaster Casts)**
    - Devices used for congenital conditions like hip dysplasia can cause pressure injuries if not carefully monitored.

Preventive Measures:

- **Frequent Skin Checks**: Regularly inspect the skin under devices to detect early signs of injury.
- **Proper Padding**: Use appropriate padding to reduce direct pressure on the skin.
- **Device Rotation**: Alternate the position of devices when possible to prevent prolonged pressure on one area.
- **Skin Protection**: Apply barrier creams or protective dressings under devices, as appropriate.
- **Correct Fit**: Ensure all devices are properly sized and secured to minimize unnecessary pressure or movement.

Implementing protocols for device management and routine skin assessment can significantly reduce the incidence of pressure injuries in neonates.

17. How can the risk of pressure injuries associated with CPAP masks be managed?

Managing the risk of pressure injuries associated with continuous positive airway pressure (CPAP) masks involves a combination of preventive measures, proper mask fitting, and regular care. Here are strategies to minimize the risk:

1. Proper Mask Selection and Fit

- **Choose the Right Mask Type**: Select a mask that suits the patient's facial structure and sleeping position (nasal masks, full-face masks, or nasal pillows).
- **Adjust the Fit**: Ensure the mask fits snugly but not too tightly. Over-tightening can lead to pressure injuries, while a loose mask may cause leaks and require frequent adjustments.
- **Use Size Guides**: Utilize manufacturer-provided size guides to ensure a proper fit.

2. Skin Protection

- **Barrier Creams or Dressings**: Apply skin-friendly barrier creams or silicone-based dressings on pressure-prone areas, such as the bridge of the nose.
- **Soft Cushions**: Use masks with built-in gel or foam cushions to reduce pressure on the skin.
- **Padding or Liners**: Add mask liners or padding to reduce friction and distribute pressure evenly.

3. Frequent Skin Checks

- **Inspect Regularly**: Monitor the skin for early signs of pressure injuries, such as redness, irritation, or breakdown, especially at contact points.
- **Rotate Pressure Points**: Encourage patients to alternate between mask types or adjust straps slightly to change pressure points.

4. Hygiene and Maintenance

- **Clean the Mask Regularly**: Ensure the mask is cleaned daily to prevent skin irritation from bacteria or oils.
- **Replace Worn Components**: Replace mask cushions, straps, and other components as they wear out to maintain proper function and fit.

5. Adjusting CPAP Settings

- **Optimize Pressure Settings**: Ensure that the CPAP pressure settings are appropriate for the patient to minimize leaks and reduce the need for overtightened masks.
- **Humidification**: Use a CPAP machine with humidification to prevent dryness, which can exacerbate skin irritation.

6. Education and Support

- **Patient Education**: Teach patients how to correctly fit and adjust their mask and recognize early signs of skin damage.
- **Follow-Up**: Schedule regular follow-ups to assess for any mask-related issues and address concerns.

7. Consider Alternative Devices

- **Explore Alternatives**: If pressure injuries persist despite preventive measures, consider alternatives such as custom-fitted masks or oral appliance therapy for suitable patients.

Implementing these strategies, along with individualized care and close monitoring, can significantly reduce the risk of pressure injuries associated with CPAP therapy.

18. What precautions should be taken to prevent pressure injuries from umbilical catheters?

Preventing pressure injuries from umbilical catheters involves careful attention to placement, maintenance, and monitoring. Here are the recommended precautions:

1. Proper Insertion and Securing of the Catheter

- **Correct placement:** Ensure the umbilical catheter is inserted to the correct depth and positioned properly to minimize tension or kinking that could lead to pressure injuries.
- **Secure fixation:** Use appropriate methods to secure the catheter to prevent dislodgement or movement, such as sterile tape or adhesive devices. Avoid overtightening, which could cause pressure on the surrounding tissue.

2. Positioning

- **Avoid tension on the catheter:** Ensure that the catheter and tubing do not exert traction on the umbilical area. This may involve using protective padding or support for the tubing.
- **Reposition the infant regularly:** Change the infant's position frequently (e.g., side-lying, supine) to redistribute pressure and avoid prolonged compression of any single area.

3. Monitoring

- **Frequent inspection:** Regularly check the insertion site and surrounding skin for early signs of pressure injuries, such as redness, blanching, or breakdown.
- **Monitor catheter function:** Ensure the catheter is functioning correctly to avoid unnecessary manipulation or reinsertion, which could increase pressure risks.

4. Use of Protective Barriers

- **Skin barriers:** Apply protective barriers (e.g., hydrocolloid or silicone dressings) around the insertion site to reduce friction and pressure.
- **Padding:** Use soft, sterile padding under the catheter tubing to protect the umbilical stump and adjacent skin from pressure.

5. Hygiene and Infection Control

- **Maintain sterility:** Ensure that the site remains clean and dry to prevent infection, which can exacerbate tissue damage.
- **Minimize handling:** Avoid unnecessary handling of the catheter and insertion site to reduce trauma.

6. Education and Team Communication

- **Educate staff:** Train healthcare providers on the correct techniques for catheter insertion, securement, and maintenance to prevent pressure injuries.
- **Team collaboration:** Ensure clear communication among the care team about catheter care plans and any concerns about pressure injuries.

7. Timely Removal

- **Assess necessity:** Remove the catheter as soon as it is no longer clinically indicated to reduce the duration of pressure risk.
- **Consider alternatives:** Evaluate if alternative vascular access methods are more appropriate for long-term use.

By implementing these measures, the risk of pressure injuries from umbilical catheters can be significantly reduced, ensuring better outcomes for neonates.

19. How can pressure injuries related to bandages and casts in neonates be prevented?

Preventing pressure injuries related to bandages and casts in neonates requires a proactive, multidisciplinary approach focused on minimizing prolonged pressure, ensuring skin integrity, and monitoring high-risk areas. Here are key strategies:

1. Skin Assessment

Frequent Checks: Inspect the skin beneath and around bandages and casts at least every shift or as clinically indicated.

Risk Identification: Identify high-risk neonates, including those with very low birth weight, prematurity, or poor perfusion.

2. Choice of Materials

Soft, Breathable Bandages: Use hypoallergenic, soft, and breathable materials that reduce friction and pressure.

Padding: Ensure adequate padding under casts or tight bandages, especially over bony prominences.

3. Proper Application

Avoid Over-Tightening: Secure bandages and casts snugly but not so tight that circulation or skin integrity is compromised.

Specialized Techniques: Use techniques like "windowing" (creating an opening in the cast over pressure-prone areas) or creating pressure-relief padding.

4. Positioning and Repositioning

Regular Position Changes: Reposition the neonate every 2–3 hours to reduce sustained pressure on one area.

Elevate Pressure Points: Use rolled towels, foam pads, or other positioning aids to elevate and cushion areas prone to pressure.

5. Moisture Management

Keep Skin Dry: Address incontinence or excessive sweating promptly to avoid skin breakdown.

Barrier Creams: Apply barrier creams to protect skin under adhesives or near bandages.

6. Monitoring and Early Intervention

Use Pressure Sensors: If available, use tools to monitor pressure under casts or bandages.

Early Removal or Adjustment: Remove or adjust any dressings, bandages, or casts showing signs of causing pressure injuries.

7. Education and Training

Train Caregivers: Educate healthcare providers and caregivers on proper application, skin assessment, and early signs of injury.

Parental Involvement: Teach parents to monitor for redness, swelling, or discomfort around dressings or casts.

8. Use of Specialized Equipment

Pressure-Relieving Surfaces: Use pressure-relieving mattresses or overlays for neonates confined to bed.

Custom-Fitted Casts: When applicable, use custom-fitted or pre-molded casts designed to distribute pressure evenly.

9. Documentation

Record Observations: Maintain clear records of skin assessments and interventions to track trends and adjust care plans accordingly.

These measures can significantly reduce the risk of pressure injuries, ensuring optimal care for neonates requiring bandages or casts.

20. How should pressure injuries related to medical devices be documented?

Pressure injuries related to medical devices should be documented thoroughly and accurately to ensure proper care, compliance with clinical guidelines, and quality improvement measures. Below are key components to include when documenting such injuries:

1. Patient Information

Name, age, and relevant medical history.

Details of current hospitalization or medical condition.

2. Device Information

Type of medical device involved (e.g., endotracheal tube, oxygen mask, urinary catheter, splint).

Location of the device on the body.

Duration of device use prior to injury.

3. Injury Description

Location: Clearly describe the anatomical site of the pressure injury.

Stage of Injury: Use standardized staging guidelines (e.g., National Pressure Injury Advisory Panel [NPIAP]) to classify the injury from Stage 1 (non-blanchable erythema) to Stage 4 (full-thickness tissue loss), or as unstageable or deep tissue pressure injury (DTPI).

Size and Depth: Measure and document the injury dimensions (length, width, depth) in centimeters.

Appearance: Note the wound bed characteristics (e.g., color, necrosis, presence of granulation tissue), surrounding skin condition, and signs of infection (e.g., erythema, warmth, purulent discharge).

4. Device Impact

Describe how the medical device caused or contributed to the pressure injury (e.g., tightness, prolonged pressure, lack of repositioning).

Include observations of device-related factors (e.g., material, fit, or positioning).

5. Timeline

Document the date and time the injury was first noted.

Note any previous signs of redness or discomfort that may have preceded the injury.

6. Interventions and Actions Taken

Adjustments made to the medical device (e.g., repositioning, padding).

Treatment interventions initiated for the pressure injury (e.g., wound dressings, debridement, antibiotics).

Preventive measures to avoid recurrence (e.g., switching to a different type of device, use of protective barriers).

7. Clinical Team Involvement

Note which healthcare professionals (e.g., wound care specialists, attending physician) were consulted or involved.

Include patient or caregiver education about managing the injury and preventing further damage.

8. Photographic Documentation (If Permitted)

Capture clear and dated photographs of the pressure injury, ensuring patient consent and adherence to hospital policies.

9. Follow-Up Plan

Schedule regular assessments to monitor the injury's progression or healing.

Document the patient's response to treatment.

10. Incident Reporting (If Required)

Complete any necessary incident reports in line with institutional policies, especially if the injury is related to improper device use or preventable factors.

By documenting these details systematically, healthcare teams can ensure continuity of care, evaluate the effectiveness of interventions, and identify opportunities for system-wide prevention strategies.

21. What are the early signs of a pressure injury in a neonate?

Early signs of a pressure injury in a neonate are subtle and may progress quickly if not addressed. Neonates are particularly vulnerable due to their delicate skin, limited mobility, and potential use of medical devices. Early signs include:

Skin Changes

1. **Non-blanchable Erythema**: Redness of the skin that does not turn white when pressed. This indicates compromised blood flow and the early stage of skin damage.
2. **Discoloration**: The skin may appear pale, dusky, or mottled in areas under pressure.
3. **Increased Warmth or Coolness**: The affected area may feel warmer or cooler than surrounding skin.
4. **Shiny or Taut Skin**: Indicates underlying edema.

Texture and Integrity

5. **Skin Indentation or Bogginess**: The area may feel softer or spongier than normal.
6. **Dryness or Cracking**: Early dehydration of the skin may indicate impending breakdown.

Behavioral Signs

7. **Pain or Discomfort**: Neonates may exhibit increased irritability, grimacing, or crying when the affected area is touched or when pressure is applied.
8. **Reluctance to Move**: A neonate might avoid moving a limb or part of the body associated with the injury due to discomfort.

Device-Related Signs

9. **Indentation from Devices**: Prolonged contact with medical devices (e.g., CPAP masks, endotracheal tubes, or IV lines) may leave marks or indentations, which can progress to injury.
10. **Moisture-Associated Skin Damage**: Excess moisture from sweat, drooling, or leakage around diapers may exacerbate skin breakdown.

Specific Locations

- **Occiput**: Back of the head, commonly affected due to prolonged supine positioning.
- **Ears**: From pressure from oxygen tubing or headgear.
- **Nares**: Related to nasal prongs or CPAP interfaces.
- **Heels and Toes**: From prolonged contact with bedding or pulse oximeter probes.

Preventive Actions

- Frequent repositioning (every 2 hours or as tolerated).
- Use of pressure-relieving devices (e.g., gel pads, foam dressings).
- Regular skin assessments by trained personnel.
- Minimizing medical device pressure by using appropriate padding or ensuring proper fit.

Early recognition and intervention are crucial to prevent progression to more severe stages of pressure injuries in neonates.

22. How is the severity of a pressure injury in a neonate assessed?

The severity of a pressure injury in a neonate is assessed using clinical staging systems adapted for their unique anatomy and physiology. The most common approach is based on the **National Pressure Injury Advisory Panel (NPIAP)** staging system, modified as needed for neonates due to their thinner, more delicate skin and specific risk factors.

Assessment Steps

1. **Inspection and Palpation:**
   - Look for areas of discoloration, erythema, or skin breakdown.
   - Palpate for induration, warmth, or bogginess, which may indicate deeper tissue injury.
2. **Staging (Adapted NPIAP Stages):**
   - **Stage 1:** Non-blanchable erythema of intact skin.
     - The skin appears red or darker than usual and does not turn white when pressed.
     - May be harder to detect in neonates due to thin skin and underdeveloped vascularity.
   - **Stage 2:** Partial-thickness skin loss with exposed dermis.
     - The injury appears as an open wound, blister, or shallow ulcer.
   - **Stage 3:** Full-thickness skin loss.
     - Fat tissue may be visible, but muscle, bone, or tendon is not exposed.
   - **Stage 4:** Full-thickness skin and tissue loss.
     - Exposed muscle, bone, or tendon, possibly with slough or eschar.
   - **Unstageable:** Obscured full-thickness skin and tissue loss.
     - Covered with slough or eschar, making the depth unclear.
   - **Deep Tissue Injury (DTI):** Persistent non-blanchable discoloration (maroon, purple) with or without intact skin.
3. **Identify Neonate-Specific Factors:**
   - **Skin Fragility:** Neonates, especially preterm infants, have underdeveloped skin layers, making them prone to epidermal stripping and injury.
   - **Pressure Points:** Focus on common pressure areas (e.g., occiput, heels, back, nose, ears) from medical devices or positioning.
   - **Medical Device-Related Pressure Injuries (MDRPIs):** Assess for injuries caused by nasal prongs, IV catheters, or pulse oximetry probes.
4. **Risk Assessment Tools:**
   - Utilize tools such as the **Braden QD Scale** (adapted for pediatrics) or institutional protocols to assess the risk of pressure injury development in neonates.

5. **Document the Injury:**
    - Describe the injury's location, size, depth, color, presence of exudate, and signs of infection (if any).
    - Take photographs (with parental consent) to monitor progression.
6. **Holistic Evaluation:**
    - Consider systemic factors like nutritional status, perfusion, and comorbidities (e.g., sepsis, hypoxia) that can worsen injury severity or delay healing.

Considerations for Neonates:

- **Accuracy of Staging:** The staging may not directly correlate with adult categories due to neonates' thinner dermis and different healing responses.
- **Parental Involvement:** Engage parents in monitoring and prevention, such as frequent repositioning and protecting vulnerable skin areas.
- **Team Approach:** Collaborate with wound care specialists, neonatologists, and nurses trained in neonatal skin care.

Early identification and accurate staging are critical for optimizing treatment and preventing complications.

23. What risk assessment tools for pressure injuries are most suitable for neonates?

Risk assessment tools for pressure injuries (PIs) in neonates need to account for their unique physiology, skin characteristics, and care environment. Below are some of the most suitable tools designed or adapted for neonates:

1. Braden Q Scale (Adapted Version for Neonates)

- **Description**: A pediatric adaptation of the Braden Scale, assessing risk based on mobility, activity, sensory perception, moisture, friction/shear, nutrition, and tissue perfusion/oxygenation.
- **Suitability for Neonates**: The Braden Q Scale has been further adapted for neonates (e.g., Braden QD Scale) by incorporating developmental factors such as gestational age and specific risks like the presence of medical devices.
- **Pros**:
    - Includes neonatal-specific criteria (e.g., immobility due to ventilators or incubators).
    - Widely validated in pediatric and neonatal populations.
- **Limitations**:
    - May require modification for extremely preterm infants.

2. Neonatal Skin Risk Assessment Scale (NSRAS)

- **Description**: Developed specifically for neonates, this tool assesses skin condition, gestational age, activity level, and medical device use.
- **Categories**:
    - General physical condition.
    - Mental state (e.g., responsiveness).

- Activity level.
- Mobility limitations.
- Nutrition.
- Skin integrity.

- **Suitability for Neonates**:
  - Explicitly designed for preterm and term neonates, including those in NICU settings.
- **Pros**:
  - Focused on neonatal-specific risks.
  - Easy to use for bedside assessments.
- **Limitations**:
  - Limited validation outside NICUs.

3. Gonzalez Risk Assessment Scale for Neonates (GRAS-N)

- **Description**: Evaluates neonates for pressure injury risk based on parameters like gestational age, weight, and use of devices.
- **Suitability for Neonates**:
  - Developed specifically for neonates with risk factors commonly encountered in NICUs.
- **Pros**:
  - Focused on neonatal physiology and environment.
- **Limitations**:
  - Less widely used and validated compared to other tools.

4. Neonatal Pressure Ulcer Risk Assessment Scale (Neonatal-PURAS)

- **Description**: A tool that assesses neonates' risk of developing pressure injuries with a focus on immobility, skin moisture, and device-related pressure.
- **Suitability for Neonates**:
  - Incorporates neonatal-specific elements such as frequent repositioning needs and sensitivity to devices.
- **Pros**:
  - Adaptable to various care settings.
- **Limitations**:
  - Requires further validation across diverse populations.

5. Neonatal Pressure Ulcer Prediction Scale (NPUPS)

- **Description**: A simplified scale assessing risk factors like gestational age, weight, immobility, and device usage.
- **Suitability for Neonates**:
  - Well-suited for preterm infants in NICUs.
- **Pros**:
  - Practical and quick to administer.
- **Limitations**:
  - Limited evidence for application in term neonates.

Choosing the Right Tool:

The choice of tool depends on the clinical setting, population, and available resources:

- **For NICUs**: NSRAS or Braden QD may be more appropriate due to their neonatal focus.
- **For General Use**: Braden QD offers a broader application across pediatric and neonatal populations.
- **For Premature Infants**: GRAS-N or NPUPS may provide a better fit due to their emphasis on low birth weight and immaturity.

Implementation Considerations:

- **Training**: Clinicians need proper training to use the chosen tool effectively.
- **Device Management**: Many neonatal PIs are device-related, so tools that emphasize this factor are essential.
- **Regular Reassessment**: Neonates' risk factors can change rapidly, necessitating frequent evaluations.

24. How frequently should high-risk neonates be monitored for pressure injuries?

High-risk neonates should be monitored **at least daily** for pressure injuries, with more frequent assessments (every 4–6 hours) in certain high-risk situations. Monitoring should be tailored based on the neonate's individual risk factors and clinical condition. Here's a guideline to consider:

1. **Baseline Risk Assessment**: Conduct an initial risk assessment using a validated tool, such as the Neonatal Skin Risk Assessment Scale (NSRAS), upon admission to the neonatal intensive care unit (NICU).
2. **Ongoing Monitoring**:
   - **Daily Skin Assessment**: Perform a thorough skin check at least once daily, focusing on areas prone to pressure injuries, such as the occiput, ears, sacrum, and heels.
   - **After Procedures or Positioning Changes**: Inspect skin following prolonged medical procedures, repositioning, or the removal of medical devices (e.g., CPAP masks, ET tubes).
3. **High-Risk Scenarios**: For neonates with specific risk factors (e.g., immobility, medical devices, edema, poor perfusion, or very low birth weight):
   - Conduct assessments every 4–6 hours, especially during the first 48 hours of using a new medical device or in situations where tissue perfusion is compromised.
4. **Intervention Documentation**:
   - Document skin condition, any pressure injury findings, and preventive measures (e.g., repositioning, use of protective dressings).

Regular, detailed monitoring helps to promptly identify early signs of pressure injuries and implement preventive measures. A multidisciplinary approach involving neonatologists, nurses, and wound care specialists ensures optimal outcomes.

25. Are there standardized protocols for monitoring pressure injuries?

Yes, there are standardized protocols for monitoring pressure injuries (PIs). These protocols are generally evidence-based and focus on assessment, documentation, prevention, and management of pressure injuries. Below are the key components commonly included in such protocols:

1. Regular Skin Assessments

- **Frequency:** Typically performed at least once per shift for hospitalized patients or as per the facility's policy.
- **Method:** Use a systematic approach, such as head-to-toe examination.
- **Risk Factors:** Assess for immobility, moisture, friction, shear, nutritional status, and medical conditions (e.g., diabetes, peripheral vascular disease).
- **Tools:** Utilize validated tools like the **Braden Scale** or **Norton Scale** to assess pressure injury risk.

2. Staging of Pressure Injuries

Follow standardized staging guidelines, such as those by the **National Pressure Injury Advisory Panel (NPIAP):**

- Stage 1: Non-blanchable erythema of intact skin.
- Stage 2: Partial-thickness skin loss with exposed dermis.
- Stage 3: Full-thickness skin loss; fat is visible.
- Stage 4: Full-thickness tissue loss; muscle, bone, or tendon exposure.
- Unstageable: Full-thickness skin/tissue loss obscured by slough or eschar.
- Deep Tissue Injury: Persistent non-blanchable deep red, maroon, or purple discoloration.

3. Documentation

- **Initial Assessment:** Document the location, size (length, width, depth), wound bed, edges, exudate, and surrounding skin condition.
- **Monitoring Changes:** Use consistent measurement tools (e.g., ruler or wound measurement apps) and photographic documentation when appropriate.
- **Frequency:** Document at every dressing change and during routine assessments.

4. Prevention Protocols

- **Repositioning:** Schedule repositioning every 2 hours or per patient tolerance.
- **Support Surfaces:** Use pressure-relieving mattresses or cushions.
- **Skin Care:** Keep skin clean and dry; use barrier creams and moisturizers.
- **Nutrition:** Ensure adequate intake of calories, protein, and hydration; consider nutritional supplementation as needed.

5. Multidisciplinary Approach

- Involve a wound care specialist, dietitian, physical therapist, and primary care provider.

- Include family or caregiver education on monitoring and prevention strategies.

6. Use of Technology

- Electronic health records (EHR) with alerts for risk assessments and follow-ups.
- Pressure-mapping systems to identify high-risk areas in bed-bound patients.

7. Education and Training

- Provide staff training on risk assessment tools, staging, and management.
- Periodic updates on new guidelines or protocols.

8. Compliance with Guidelines

Adhere to recommendations from organizations such as:

- **National Pressure Injury Advisory Panel (NPIAP)**
- **European Pressure Ulcer Advisory Panel (EPUAP)**
- **Agency for Healthcare Research and Quality (AHRQ)**

If you are part of a healthcare facility, standardized protocols might be tailored to local resources, patient demographics, and care settings, but adherence to the above frameworks ensures comprehensive and effective pressure injury management.

26. What treatments are most appropriate for superficial pressure injuries in neonates?

Superficial pressure injuries in neonates require meticulous care to promote healing, prevent infection, and minimize discomfort. The treatment plan should consider the neonate's fragile skin, limited ability to tolerate adhesives, and increased susceptibility to infection. Here are the most appropriate treatments:

1. Wound Cleansing

- **Clean the wound gently:** Use sterile normal saline or a mild, non-cytotoxic wound cleanser to clean the area. Avoid antiseptics like iodine or hydrogen peroxide, which can damage delicate tissues.
- **Avoid vigorous scrubbing:** Pat gently to clean without causing further trauma.

2. Protect the Wound

- **Use appropriate dressings:** Select dressings that maintain a moist wound environment and protect the injury:
  - **Hydrogel dressings**: For dry superficial injuries.
  - **Foam dressings**: To cushion and absorb exudate in slightly exuding wounds.
  - **Transparent film dressings**: For shallow wounds with minimal drainage, allowing visualization of the site.

- **Avoid adhesive tapes:** Use silicone-based or low-adherent dressings to prevent further skin damage during dressing changes.

3. Optimize Skin Care

- **Protect surrounding skin:** Apply a barrier cream or ointment (e.g., zinc oxide or petrolatum) to the periwound area to prevent maceration.
- **Minimize friction and shear:** Reposition the neonate frequently and use soft bedding or positioning devices to distribute pressure.

4. Manage Pain

- Neonates may not verbally express pain, so consider signs like restlessness or crying.
- Use non-pharmacological interventions (e.g., swaddling, skin-to-skin contact) and, if needed, consult a pediatrician for safe analgesics.

5. Prevent Infection

- Keep the area clean and dry.
- Monitor for signs of infection (e.g., redness, swelling, purulent discharge) and initiate topical or systemic antibiotics if needed, based on clinical findings.

6. Optimize Nutrition and Hydration

- Ensure adequate nutritional support to promote wound healing.
- Address any feeding challenges to provide sufficient protein, vitamins (e.g., A, C), and zinc.

7. Repositioning and Pressure Relief

- **Reposition frequently:** Change the neonate's position at least every 2 hours.
- Use gel pads, foam mattresses, or pressure-relief devices to reduce pressure on bony prominences.

8. Specialized Care

- For worsening wounds or those failing to heal, consult a pediatric wound care specialist.
- Advanced therapies like negative pressure wound therapy (NPWT) are rarely indicated in neonates but may be considered in severe cases under expert guidance.

Key Considerations

- **Parental education:** Teach parents or caregivers how to recognize signs of deterioration and maintain skin care at home.
- **Multidisciplinary care:** Collaborate with neonatologists, nurses, and wound care specialists for comprehensive management.

Prompt intervention and attention to preventive measures are critical to achieving optimal outcomes for superficial pressure injuries in neonates.

27. How are advanced-stage pressure injuries managed in neonates?

Managing advanced-stage pressure injuries in neonates requires a delicate and multidisciplinary approach due to their fragile skin, immature immune system, and unique physiological considerations. The management strategy focuses on minimizing further injury, promoting healing, and preventing complications. Here's an overview:

1. Assessment and Staging

- Use an appropriate pressure injury staging system (e.g., NPIAP) adapted for neonates.
- Assess the size, depth, and extent of tissue damage, as well as signs of infection (redness, warmth, purulent discharge, or systemic signs such as fever).

2. Pressure Relief and Prevention of Further Injury

- **Pressure Redistribution**: Use pressure-redistributing devices, such as gel pads, foam overlays, or specialized mattresses.
- **Frequent Repositioning**: Reposition neonates every 2-3 hours while ensuring careful handling to prevent friction and shear forces.
- **Skin Protection**: Apply gentle, protective barriers (zinc oxide or silicone-based products) to shield the skin around the wound and prevent maceration.

3. Wound Care

- **Cleansing**: Clean the wound with sterile saline or an appropriate wound cleanser. Avoid harsh antiseptics that can damage healthy tissue.
- **Debridement**: If necrotic tissue is present, opt for gentle autolytic or enzymatic debridement, as sharp debridement may not be suitable for neonates.
- **Dressings**:
  - Use moisture-retentive dressings (e.g., hydrocolloid, foam, or silicone dressings) to maintain an optimal wound-healing environment.
  - Avoid dressings with adhesive that might harm delicate neonatal skin.
  - Consider antimicrobial dressings if infection is present or suspected.
- **Monitoring**: Check the dressing frequently for saturation or leakage, ensuring the wound remains clean and moist.

4. Infection Control

- Monitor for local or systemic signs of infection.
- **Topical Antibiotics**: Use if there are localized signs of infection, but avoid indiscriminate use to prevent resistance.
- **Systemic Antibiotics**: Consider for systemic infection or if there are signs of cellulitis, osteomyelitis, or sepsis.

5. Nutritional Support

- Adequate nutrition is critical for wound healing. Ensure the neonate receives sufficient protein, calories, vitamins (e.g., A, C), and minerals (e.g., zinc).
- Fortify feeds or consider parenteral nutrition if oral intake is inadequate.

6. Multidisciplinary Involvement

- Involve specialists such as neonatologists, wound care nurses, nutritionists, and physiotherapists.
- Consider consulting a pediatric dermatologist or surgeon for complex or non-healing wounds.

7. Parental/Caregiver Education

- Educate parents on repositioning techniques, pressure relief strategies, and the importance of maintaining a clean and supportive environment.
- Provide emotional support as dealing with pressure injuries in neonates can be distressing for caregivers.

8. Monitoring and Follow-Up

- Regularly reassess the wound for healing progression or complications.
- Adjust the management plan based on the response to treatment and any changes in the neonate's condition.

9. Surgical Intervention

- Reserved for severe cases (e.g., Stage 4 injuries with exposed bone or deep tissue loss) that do not respond to conservative treatment.
- Procedures such as flap reconstruction might be considered, but only under expert guidance and with careful consideration of risks.

Key Considerations

- **Fragility of Neonatal Skin**: Ensure all interventions are as non-invasive and gentle as possible.
- **Preventative Strategies**: Focus heavily on preventing pressure injuries through proper positioning, skin care, and risk assessments using tools like the Braden Q scale modified for neonates.

Advanced-stage pressure injury management in neonates is a delicate process that prioritizes the neonate's safety, comfort, and healing potential while preventing long-term complications.

28. Is a multidisciplinary approach necessary for the treatment of neonatal pressure injuries?

Yes, a **multidisciplinary approach** is crucial for the effective prevention, management, and treatment of **neonatal pressure injuries**. These injuries, though relatively uncommon, can have significant impacts on the health and well-being of neonates, particularly those in intensive care settings. The complexity of their care requires the collaboration of various healthcare professionals, each bringing unique expertise to ensure optimal outcomes.

Why a Multidisciplinary Approach Is Necessary:

1. **Holistic Assessment and Care Planning**:
   - Neonatal pressure injuries often result from prolonged immobility, medical devices, or fragile skin. Each factor requires input from different specialists to address comprehensively.
   - Regular skin assessments by **neonatologists**, **nurses**, and **wound care specialists** are essential for early detection and prevention.
2. **Customized Treatment**:
   - **Neonatologists** and **pediatricians**: Lead the overall care, ensuring systemic conditions such as poor perfusion or malnutrition are addressed.
   - **Nurses**: Play a frontline role in daily skin care, repositioning schedules, and medical device adjustments to reduce pressure.
   - **Wound care specialists**: Provide expertise in dressing selection, wound debridement, and advanced therapies if necessary.
   - **Nutritionists**: Ensure adequate protein and micronutrient intake for wound healing and skin integrity.
   - **Physical and occupational therapists**: Recommend positioning techniques to minimize pressure and improve circulation.
3. **Prevention Strategies**:
   - Collaboration among **respiratory therapists**, **biomedical engineers**, and **nurses** can optimize medical device placement and minimize pressure from equipment like CPAP masks or IV tubing.
   - Involvement of **infection control specialists** helps reduce the risk of secondary infections in pressure injuries.
4. **Family Involvement**:
   - Educating families about proper handling, positioning, and signs of pressure injuries is critical. Multidisciplinary teams ensure consistent communication with families.
5. **Quality Improvement and Research**:
   - Regular reviews of cases by a multidisciplinary team can identify risk factors and gaps in care, leading to the development of prevention protocols.

Benefits of a Multidisciplinary Approach:

- **Improved outcomes**: Enhanced healing rates and prevention of complications.
- **Comprehensive care**: Addressing both local wound care and systemic issues.
- **Education**: Consistent training for staff and families, ensuring adherence to best practices.

In summary, neonatal pressure injuries are multifactorial in origin and management, necessitating the expertise of a multidisciplinary team. This collaborative approach is vital for achieving the best outcomes for this vulnerable population.

29. What dressings are recommended for neonates with fragile skin?

When selecting dressings for neonates with fragile skin, special care is required to minimize the risk of skin damage and to promote healing. The following dressings are recommended:

1. Silicone-Based Dressings

- **Examples**: Mepitel®, Mepilex®, Allevyn Gentle®
- **Benefits**:
  - Gentle adhesion that minimizes trauma during removal.
  - Suitable for delicate neonatal skin.
  - Maintains a moist wound environment to support healing.

2. Hydrocolloid Dressings

- **Examples**: Duoderm®, Tegaderm® Hydrocolloid
- **Benefits**:
  - Adhere gently to the skin.
  - Provide a moist healing environment.
  - Ideal for low-exudate wounds.

3. Hydrogel Dressings

- **Examples**: Aquaflo®, IntraSite® Gel
- **Benefits**:
  - Hydrate dry wounds.
  - Reduce pain during dressing changes.
  - Do not stick to the wound bed.

4. Foam Dressings

- **Examples**: Allevyn®, Polymem®, Mepilex Lite®
- **Benefits**:
  - Provide cushioning to protect the wound.
  - Absorb exudate without adhering to fragile skin.
  - Good for wounds with moderate exudate.

5. Transparent Film Dressings

- **Examples**: Tegaderm®, Opsite Flexigrid®
- **Benefits**:
  - Protect intact skin and superficial wounds.
  - Allow visualization of the wound or IV site.

- Easy to apply and remove with minimal trauma.

6. Non-Adherent Dressings

- **Examples**: Telfa®, Adaptic Touch®, Vaseline Gauze®
- **Benefits**:
  - Prevent adherence to the wound bed.
  - Minimize skin trauma during dressing changes.
  - Ideal for superficial wounds.

7. Soft Silicone Tape

- **Examples**: Mepitac®, Siltape®
- **Benefits**:
  - Secures dressings without causing skin damage.
  - Reusable and repositionable.

General Considerations for Neonates:

- Avoid adhesives wherever possible; if required, use barrier films like Cavilon® or Silesse® under adhesives to reduce skin damage.
- Change dressings only when necessary to minimize handling and reduce stress.
- Maintain a gentle, atraumatic approach to application and removal.

Key Practices:

- Use warm hands to reduce discomfort during dressing application.
- Ensure the dressing size is appropriate for the wound.
- Keep the surrounding skin clean and dry to prevent maceration.

Careful selection of dressing materials and techniques can significantly improve outcomes and prevent complications in neonates with fragile skin.

30. How are advanced dressings for pressure injuries used in neonatal care?

Advanced dressings are widely used in neonatal care for managing **pressure injuries** (PIs) due to their ability to provide optimal wound healing conditions while minimizing trauma to delicate neonatal skin. These dressings are chosen carefully to address the unique needs of neonates, such as their thin skin, limited ability to regulate temperature, and higher risk of infection. Here's an overview of how advanced dressings are used in neonatal care:

Types of Advanced Dressings and Their Applications

1. **Hydrocolloids**
   - **Function**: Provide a moist wound environment and are effective for managing mild to moderate exudate.

- **Use in Neonates**: These dressings are often used for superficial PIs or partial-thickness wounds. They adhere well to neonatal skin but require careful removal to prevent skin stripping.

2. **Hydrogels**
   - **Function**: Rehydrate dry wounds and provide moisture, promoting autolytic debridement.
   - **Use in Neonates**: Ideal for dry or necrotic PIs with minimal exudate. Hydrogels are gentle and soothing, making them suitable for fragile neonatal skin.
3. **Foam Dressings**
   - **Function**: Absorb moderate to heavy exudate while providing cushioning and thermal insulation.
   - **Use in Neonates**: Applied to protect bony prominences or absorb exudate from more severe PIs. Non-adhesive foams are preferred to prevent skin damage.
4. **Silicone Adhesive Dressings**
   - **Function**: Minimize pain and trauma during dressing changes while maintaining a moist environment.
   - **Use in Neonates**: Commonly used to prevent and treat PIs, especially on fragile skin, such as around medical devices or at pressure points.
5. **Transparent Film Dressings**
   - **Function**: Protect the wound from external contaminants while allowing moisture vapor exchange.
   - **Use in Neonates**: Typically used as a secondary dressing or for superficial, non-exudative wounds. These are also used to secure medical devices without causing irritation.
6. **Alginate Dressings**
   - **Function**: Manage moderate to heavy exudate and promote hemostasis.
   - **Use in Neonates**: Less commonly used due to the risk of over-drying the wound bed but may be used for heavily exudating wounds under supervision.
7. **Antimicrobial Dressings**
   - **Function**: Deliver antimicrobial agents (e.g., silver, honey) to prevent or treat infection.
   - **Use in Neonates**: Used sparingly and cautiously due to potential systemic absorption of antimicrobial agents.

Considerations for Use in Neonates

1. **Skin Integrity**: Neonatal skin is extremely fragile, so non-adhesive or silicone-backed dressings are preferred to minimize trauma during application and removal.
2. **Thermoregulation**: Dressings that provide thermal insulation are favored to help neonates maintain body temperature.
3. **Exudate Management**: The amount of exudate must be carefully monitored, as excessive moisture can lead to maceration, while insufficient moisture can delay healing.
4. **Risk of Infection**: Neonates are at higher risk of infection, so dressings with antimicrobial properties may be used in infected or high-risk wounds, with strict monitoring.
5. **Medical Device-Associated PIs**: Advanced dressings are frequently applied to prevent or treat PIs caused by medical devices such as endotracheal tubes, CPAP masks, or IV catheters.

Techniques for Application

- **Skin Preparation**: Clean the wound gently with saline or an appropriate cleanser to prevent further damage.
- **Dressing Selection**: Choose the smallest size that adequately covers the wound to minimize exposure to adhesives.
- **Securement**: Use secondary fixation methods (e.g., soft bandages) instead of adhesive tapes when possible.
- **Monitoring**: Inspect the wound and surrounding skin regularly for signs of infection, maceration, or dressing-related irritation.

Prevention Strategies with Advanced Dressings

- Preventive use of foam or silicone dressings on high-risk areas (e.g., occiput, sacrum, heels) to distribute pressure and reduce shear.
- Use of transparent film or hydrocolloid dressings under medical devices to mitigate device-related PIs.

Conclusion

Advanced dressings play a crucial role in managing and preventing pressure injuries in neonatal care. The choice of dressing is tailored to the wound characteristics and the neonate's specific needs. The primary goals are to support healing, minimize pain, and protect the fragile neonatal skin while closely monitoring for any adverse effects.

31. What are the most common complications of neonatal pressure injuries?

Neonatal pressure injuries (PIs) can lead to several complications, which can significantly impact the health and well-being of newborns. The most common complications include:

1. Infection

- **Localized Infection**: Pressure injuries can lead to cellulitis or wound infection due to bacterial colonization.
- **Systemic Infection**: In severe cases, untreated or poorly managed pressure injuries can result in bacteremia or sepsis, especially in neonates with immature immune systems.

2. Delayed Healing

- Neonates often have reduced skin integrity and healing capacity compared to older children and adults, which can prolong recovery and increase the risk of complications.

3. Pain and Discomfort

- Neonates may experience pain and stress due to pressure injuries, which can negatively affect feeding, sleep, and overall development.

4. Scarring

- As the skin heals, hypertrophic scars or contractures can develop, potentially leading to functional impairments or cosmetic issues.

5. Impaired Skin Barrier Function

- Pressure injuries can disrupt the skin's protective barrier, increasing susceptibility to fluid loss, temperature instability, and secondary infections.

6. Tissue Necrosis

- Prolonged pressure can lead to ischemia and necrosis of the affected tissue, making treatment more complex and increasing the risk of infection.

7. Impact on Neurodevelopment

- Chronic pain, stress, or systemic complications like sepsis from pressure injuries can negatively affect a neonate's neurodevelopmental outcomes.

8. Psychosocial and Emotional Impact on Families

- Families may experience stress, anxiety, and guilt over the occurrence and management of neonatal pressure injuries, impacting parental bonding and well-being.

9. Increased Healthcare Utilization

- Complications often require prolonged hospital stays, additional interventions (e.g., antibiotics, surgical debridement), and increased healthcare costs.

Prevention and Early Intervention

Preventing pressure injuries in neonates is critical. This includes:

- Frequent repositioning and pressure relief.
- Proper use and placement of medical devices.
- Skin barrier protection strategies.
- Regular skin assessments and early treatment of any developing injuries.

Neonatal pressure injuries, though preventable, require diligent monitoring and care to minimize complications.

32. Can pressure injuries lead to systemic infections in neonates?

Yes, pressure injuries in neonates can potentially lead to systemic infections. While neonates are generally at lower risk for pressure injuries compared to adults, those who are critically ill or require prolonged hospitalization are vulnerable due to factors such as immobility, fragile skin, and the use of medical devices (e.g., nasal cannulas, endotracheal tubes). When a pressure injury develops, it compromises the integrity of the skin, which is a primary barrier against infection. If not properly managed, bacteria can invade the injured area, leading to local infections that may progress to systemic infections, including **sepsis**.

Mechanisms and Risk Factors

1. **Skin Integrity Compromise**:
   - Neonatal skin is thinner and less keratinized, providing less protection against bacterial entry.
   - Pressure injuries, especially those with open or necrotic tissue, are susceptible to colonization by pathogens like **Staphylococcus aureus**, **Klebsiella spp.**, or **Pseudomonas aeruginosa**.
2. **Immature Immune System**:
   - Neonates have an underdeveloped immune response, making them less capable of containing local infections.
3. **Medical Device-Related Pressure Injuries**:
   - Injuries caused by devices such as CPAP prongs, pulse oximeters, or intravenous lines can serve as direct pathways for infection.
4. **Co-Morbid Conditions**:
   - Prematurity, low birth weight, or the presence of invasive lines (e.g., central venous catheters) increase the risk of systemic infection.

Clinical Implications

- **Local Infection**: Signs include erythema, purulent discharge, or foul odor at the pressure injury site.
- **Systemic Infection**: Signs include fever, hypothermia, tachycardia, hypotension, or other markers of sepsis (e.g., elevated CRP or procalcitonin).
- **Complications**: If untreated, systemic infections can result in bacteremia, septic shock, or multi-organ dysfunction.

Management Strategies

1. **Prevention**:
   - Regular repositioning to reduce pressure points.
   - Use of pressure-relieving surfaces (e.g., gel pads or silicone dressings).
   - Gentle handling to minimize friction or shear forces on the skin.
2. **Early Detection**:
   - Vigilant monitoring of high-risk areas (e.g., occiput, sacrum, heels).
   - Immediate identification of signs of local or systemic infection.
3. **Treatment**:
   - **Local Care**: Clean the wound, apply appropriate dressings, and manage exudate.
   - **Systemic Antibiotics**: Initiate based on suspected or confirmed bacterial pathogens in cases of systemic infection.

- **Supportive Care**: Ensure adequate nutrition, hydration, and oxygenation to promote wound healing and combat infection.

In summary, while pressure injuries are not a common source of systemic infections in neonates, they can lead to severe complications if not promptly recognized and managed. Prevention and meticulous care of pressure-prone areas are critical in mitigating these risks.

33. What are the signs of infection in a neonatal pressure injury?

In neonates, pressure injuries can be particularly challenging to assess for infection due to their immature immune systems and the subtle presentation of symptoms. Here are common **signs of infection in a neonatal pressure injury**:

Local Signs

1. **Increased Erythema**: Redness around the wound extending beyond the margin of the pressure injury.
2. **Swelling or Induration**: Swelling that may feel firm or hard to the touch.
3. **Warmth**: The area around the pressure injury feels warmer than the surrounding skin.
4. **Purulent Discharge**: The presence of pus or cloudy, foul-smelling exudate from the wound.
5. **Delayed Healing**: A wound that does not show signs of improvement over time.
6. **Pain or Tenderness**: Increased sensitivity in the area, although this may be challenging to assess in non-verbal neonates.
7. **Necrosis**: Tissue breakdown or darkened areas indicating possible infection-related tissue death.

Systemic Signs

1. **Fever or Hypothermia**: Elevated or subnormal body temperature, as neonates may not mount a robust febrile response.
2. **Lethargy or Irritability**: Unusual tiredness or difficulty consoling the neonate.
3. **Poor Feeding**: Decreased interest in feeding or difficulty feeding.
4. **Tachycardia or Bradycardia**: Abnormal heart rate patterns may indicate systemic involvement.
5. **Respiratory Distress**: Increased respiratory rate or effort.

Laboratory and Diagnostic Indicators

1. **Elevated White Blood Cell Count (WBC)**: Suggestive of infection, though neonatal immune responses may vary.
2. **C-Reactive Protein (CRP) Elevation**: Often used as a marker for systemic infection.
3. **Positive Culture**: Bacterial growth from a wound swab or blood culture confirming infection.
4. **Elevated Procalcitonin**: May indicate systemic bacterial infection.

Special Considerations

- Neonates are at higher risk for **sepsis** with localized infections, and even mild signs should prompt thorough evaluation.
- Always monitor for signs of infection spreading, such as **cellulitis**, lymphangitis, or worsening systemic symptoms.

Management

If infection is suspected, treatment typically includes:

- **Topical antibiotics** for localized infection.
- **Systemic antibiotics** for more significant or systemic infection.
- **Wound care** to prevent further complications and promote healing.

Close monitoring and consultation with a pediatric infectious diseases specialist are recommended in severe or unclear cases.

34. Do pressure injuries affect normal skin development in neonates?

Pressure injuries in neonates can indeed affect normal skin development, as the skin of neonates, particularly preterm infants, is thinner, less keratinized, and more fragile compared to that of older children and adults. Here's how pressure injuries may impact normal skin development:

1. Delayed Skin Maturation

- Pressure injuries can disrupt the normal progression of skin development, particularly in preterm neonates who are still in the process of skin maturation. Damage to the dermal and epidermal layers may slow the natural development of the stratum corneum and other skin structures.

2. Increased Risk of Scarring

- Neonatal skin has a greater regenerative capacity but is also more prone to scarring. Pressure injuries that extend into deeper layers of the skin can result in scar formation, which can affect the normal appearance and functionality of the skin.

3. Compromised Skin Integrity

- Pressure injuries can lead to compromised skin integrity, making the skin more susceptible to infections and further damage. This may alter the normal protective barrier function of the skin.

4. Changes in Skin Properties

- Chronic pressure injuries may lead to changes in skin elasticity, thickness, and pigmentation over time, potentially impacting the overall development of the affected skin area.

5. Impact on Skin Appendages

- Damage from pressure injuries may disrupt the normal development of skin appendages like sweat glands, sebaceous glands, and hair follicles, especially if the injury affects the dermis where these structures reside.

6. Potential Systemic Effects

- Severe or repeated injuries can lead to chronic inflammation, which may have systemic effects, potentially influencing overall growth and skin development.

Prevention and Management

To minimize the impact of pressure injuries on skin development in neonates:

- **Frequent Position Changes:** Rotate the infant's position every 2–4 hours to relieve pressure.
- **Use Support Surfaces:** Utilize pressure-redistributing mattresses and cushions.
- **Skin Care Protocols:** Maintain proper hydration and use emollients to enhance skin barrier function.
- **Monitor Closely:** Early detection of redness or skin changes can prevent the progression to deeper injuries.
- **Nutrition:** Ensure adequate nutrition to support skin healing and development.

While pressure injuries can potentially affect normal skin development, proactive measures and prompt treatment can significantly mitigate these effects.

35. What are the long-term outcomes of neonatal pressure injuries?

The long-term outcomes of neonatal pressure injuries can vary depending on the severity, location, and timely management of the injury. Below are the potential long-term consequences:

1. Scarring and Cosmetic Concerns

- Deep or severe pressure injuries can result in permanent scarring, which may be cosmetically concerning, especially if located on visible areas such as the face.
- Keloid or hypertrophic scar formation may occur, particularly in neonates predisposed to such conditions due to genetic factors.

2. Functional Impairment

- If pressure injuries occur over joints or other critical areas, they may lead to contractures, restricted mobility, or reduced functionality.
- Pressure injuries affecting sensory organs (e.g., eyes, ears) can cause sensory deficits or deformities.

3. Chronic Wounds or Recurrent Skin Breakdown

- Poor healing or chronic wounds can develop in neonates with severe or improperly managed injuries.
- Areas with previous pressure injuries may have thinner or less resilient skin, predisposing them to future breakdowns.

4. Psychological and Developmental Impact

- Visible scars or deformities may lead to psychological distress or social challenges as the child grows older.
- Pain or discomfort during wound healing in infancy may have subtle effects on neurodevelopment or pain perception, although evidence is limited.

5. Infection-Related Complications

- Pressure injuries that become infected can lead to long-term complications such as osteomyelitis or sepsis, potentially affecting overall growth and development.
- Neonates who experience systemic infections may have residual health issues.

6. Impact on Growth

- Severe injuries may impair growth in the affected area, particularly in cases involving bones or soft tissue near growth plates.

7. Parental and Caregiver Impact

- Neonatal pressure injuries can lead to long-term stress, guilt, or anxiety for parents or caregivers, potentially affecting family dynamics and the child's care environment.

Prevention and Follow-Up

Effective prevention, early detection, and appropriate management of neonatal pressure injuries are crucial to minimizing long-term complications. Regular follow-up with dermatologists, pediatric surgeons, or other specialists may be necessary to monitor for and address any residual effects. Additionally, addressing cosmetic and functional issues early, including through physical therapy or reconstructive surgery, can improve outcomes.

36. What is the role of the nurse in preventing neonatal pressure injuries?

The role of the nurse in preventing neonatal pressure injuries is critical and multifaceted, given the vulnerability of neonates to skin damage due to their underdeveloped skin and prolonged exposure to medical devices. Here are the key responsibilities:

1. Assessment and Monitoring

- **Initial Skin Assessment:** Perform a comprehensive skin assessment on admission and regularly thereafter, using standardized tools such as the Neonatal Skin Condition Score (NSCS).
- **Risk Identification:** Identify neonates at risk for pressure injuries, such as those with prematurity, low birth weight, or requiring prolonged use of medical devices (e.g., CPAP, ET tubes, or IV lines).

2. Skin Care and Maintenance

- **Gentle Skin Care:** Use mild cleansers and avoid harsh scrubbing to protect the fragile neonatal skin.
- **Moisturization:** Maintain adequate skin hydration by applying appropriate emollients, particularly in neonates with dry or compromised skin.
- **Minimize Adhesive Use:** Reduce the use of adhesive products or use alternatives like hydrocolloid dressings to prevent skin tears when securing devices.

3. Positioning and Repositioning

- **Frequent Repositioning:** Regularly reposition neonates to redistribute pressure and minimize prolonged pressure on specific areas.
- **Use of Support Surfaces:** Employ soft, pressure-relieving mattresses, gel pads, or cushions to reduce the risk of pressure injuries.

4. Device Management

- **Proper Fitting of Devices:** Ensure that medical devices like CPAP masks or nasal cannulas are appropriately sized and adjusted to avoid excessive pressure on the skin.
- **Frequent Inspection:** Regularly inspect areas in contact with medical devices for early signs of pressure injury, such as redness or skin breakdown.
- **Secure Devices Appropriately:** Use non-adhesive or low-tension methods to secure devices to prevent undue stress on the skin.

5. Education and Advocacy

- **Educate Staff and Families:** Teach healthcare providers and parents about the importance of skin care and the early signs of pressure injuries.
- **Advocate for Policies:** Support the development and implementation of protocols for neonatal skin care and pressure injury prevention in the clinical setting.

6. Documentation and Communication

- **Accurate Documentation:** Record skin assessments, preventive measures taken, and any signs of pressure injury in the neonatal medical record.
- **Interdisciplinary Communication:** Collaborate with the healthcare team, including physicians, wound care specialists, and physical therapists, to develop and implement a care plan for at-risk neonates.

7. Quality Improvement

- **Monitor Outcomes:** Participate in monitoring and analyzing rates of neonatal pressure injuries to identify trends and areas for improvement.
- **Participate in Training:** Stay updated on evidence-based practices and advances in neonatal skin care through continuous education and training.

By diligently performing these responsibilities, nurses play a pivotal role in minimizing the incidence of neonatal pressure injuries, promoting skin integrity, and enhancing the overall outcomes for neonates in their care.

37. What specific competencies should a nurse have to manage pressure injuries in neonates?

Managing pressure injuries in neonates requires specialized competencies due to their unique physiology, fragile skin, and vulnerability to complications. The following are specific competencies a nurse should have:

Clinical Knowledge and Understanding

1. **Skin Anatomy and Physiology in Neonates**:
   - Understand the structural differences between neonatal and adult skin, including its thinner epidermis and reduced collagen and elastin content.
   - Recognize the increased risk of injury from shear, friction, and pressure.
2. **Risk Factors for Pressure Injuries**:
   - Identify neonate-specific risk factors, such as immobility, medical devices (e.g., oxygen masks, nasogastric tubes, IV lines), and critical illness.
   - Understand the role of comorbidities like prematurity, low birth weight, and poor nutrition.
3. **Pressure Injury Staging**:
   - Be proficient in staging pressure injuries using appropriate guidelines (e.g., NPUAP/EPUAP staging).
   - Differentiate between pressure injuries and other skin conditions, such as diaper dermatitis or skin tears.

Assessment Skills

4. **Skin and Risk Assessments**:
   - Conduct systematic skin assessments at regular intervals.

- Use validated tools for neonatal pressure injury risk assessment (e.g., Braden QD scale adapted for neonates).

5. **Device-Related Pressure Injury Monitoring**:
    - Monitor areas in contact with medical devices for early signs of pressure injury.
    - Assess skin under adhesives, such as tape or monitoring electrodes, for integrity.

Intervention and Prevention

6. **Positioning and Repositioning Techniques**:
    - Employ gentle handling and frequent repositioning strategies to minimize pressure and shear forces.
    - Use positioning aids suitable for neonates, such as gel pads and foam cushions.
7. **Use of Protective Devices and Dressings**:
    - Select and apply pressure-relieving dressings, such as silicone-based or hydrocolloid dressings, as indicated.
    - Appropriately secure medical devices to prevent pressure without compromising their function.
8. **Nutritional Support**:
    - Collaborate with dietitians to ensure optimal nutrition to promote skin healing and prevent further injury.

Wound Care Management

9. **Management of Pressure Injuries**:
    - Demonstrate proficiency in cleaning, dressing, and managing neonatal wounds.
    - Select appropriate wound care products based on the stage of the injury and the neonate's condition.
10. **Infection Prevention**:
    - Apply strict aseptic techniques during wound care to prevent infections.
    - Recognize signs of local or systemic infection and escalate care appropriately.

Education and Communication

11. **Family Education**:
    - Educate caregivers on the importance of pressure injury prevention and care.
    - Involve families in repositioning and skin care practices when feasible.
12. **Interdisciplinary Collaboration**:
    - Work closely with neonatologists, wound care specialists, and other healthcare providers to develop and implement individualized care plans.

Quality Improvement and Research

13. **Protocol Adherence**:
    - Adhere to evidence-based guidelines and hospital protocols for pressure injury prevention and management.
14. **Data Collection and Reporting**:
    - Document assessments, interventions, and outcomes accurately.
    - Participate in quality improvement initiatives aimed at reducing pressure injury incidence in neonates.

15. **Continuous Education**:
    - Stay updated on advances in neonatal wound care and pressure injury management through ongoing education and training.

By mastering these competencies, nurses can effectively prevent and manage pressure injuries in neonates, improving outcomes and quality of care.

38. How should pressure injuries in neonates be documented properly?

Proper documentation of pressure injuries in neonates is essential for clinical management, quality improvement, and compliance with healthcare standards. Here is how pressure injuries in neonates should be documented:

1. Initial Assessment

- **Patient Information**: Include the neonate's name, medical record number, gestational age, birth weight, and current weight.
- **Date and Time of Assessment**: Document when the injury was first identified.
- **Location**: Specify the anatomical site of the pressure injury (e.g., occiput, sacrum, elbows, heels).
- **Description of Skin Lesion**:
    - Size (length, width, and depth in millimeters or centimeters).
    - Color (e.g., erythematous, pale, purple, black).
    - Wound edges (e.g., well-defined, irregular).
    - Presence of undermining or tunneling.
    - Exudate (type, amount, and color, e.g., serous, purulent).
    - Odor (if present).
- **Stage of Pressure Injury** (based on National Pressure Injury Advisory Panel guidelines):
    - Stage 1: Non-blanchable erythema.
    - Stage 2: Partial-thickness skin loss.
    - Stage 3: Full-thickness skin loss.
    - Stage 4: Full-thickness skin and tissue loss.
    - Unstageable: Obscured by slough/eschar.
    - Deep Tissue Pressure Injury (DTPI): Persistent discoloration.

2. Contributing Factors

- **Medical Devices**: Note if the injury is associated with a medical device (e.g., CPAP masks, IV tubing, pulse oximeters).
- **Risk Factors**:
    - Prematurity.
    - Fragile or underdeveloped skin.
    - Edema or hypoalbuminemia.
    - Immobility or prolonged positioning.

3. Interventions

- **Treatment Initiated**:

- Wound care measures (e.g., application of dressings, barrier creams).
- Offloading devices (e.g., gel pads, repositioning schedules).
- Nutritional support.

- **Changes to Care Plan**: Document any adjustments made to prevent further injury, such as device padding or repositioning frequency.

4. Photographic Evidence

- With parental consent, capture high-quality photographs to support written documentation. Ensure the date and patient identifiers are not included in the image to maintain confidentiality.

5. Progress Notes

- **Frequency of Monitoring**: Daily or per institutional policy.
- **Wound Healing Progress**: Note changes in size, appearance, or signs of infection.
- **Team Involvement**: Include consultations with wound care specialists, dermatologists, or neonatologists.

6. Parental Communication

- Document discussions with parents regarding the injury, its implications, and the care plan.

7. Quality Improvement

- Report the pressure injury to the hospital's quality improvement team or risk management as required.
- Contribute to institutional databases for neonatal pressure injury tracking and prevention strategies.

By adhering to these documentation guidelines, clinicians can ensure thorough assessment and management of neonatal pressure injuries, promoting better outcomes and compliance with care standards.

39. What educational tools can be used to train nurses about pressure injuries?

Training nurses about pressure injuries requires a blend of theoretical knowledge and practical skills. Here are some effective educational tools and strategies:

1. Interactive E-Learning Modules

- Online platforms can provide comprehensive, self-paced training on the causes, prevention, staging, and management of pressure injuries.
- Modules can include quizzes, animations, and case studies to enhance engagement.

2. Simulation Training

- Use high-fidelity mannequins or wound simulators to replicate real-life scenarios.
- Allow nurses to practice wound assessment, cleaning, and dressing applications.

3. Wound Care Workshops

- Hands-on workshops can focus on identifying pressure injuries, staging them, and applying evidence-based interventions.
- Include interactive sessions with wound care experts for Q&A.

4. Case Studies and Group Discussions

- Analyze real-life cases or hypothetical scenarios to understand complex situations.
- Encourage group discussions to develop collaborative problem-solving skills.

5. Mobile Apps

- Apps such as **WoundRounds** or **PUPPI (Pressure Ulcer Prevention Program Initiative)** provide guidance on pressure injury staging, prevention protocols, and documentation.

6. Visual Aids

- Use posters, infographics, and charts showing the different stages of pressure injuries and corresponding treatments.
- Display them in staff rooms and clinical areas.

7. Role-Playing

- Nurses can role-play patient care scenarios, including turning schedules, skin inspections, and patient education.

8. Gamification

- Incorporate game-based learning, such as virtual challenges or board games, to make training engaging.
- For instance, quiz-style games on pressure injury prevention can reinforce knowledge.

9. Virtual Reality (VR)

- VR systems can immerse nurses in simulated environments where they assess and treat virtual patients with pressure injuries.

10. Educational Videos

- Use professionally made videos to demonstrate correct positioning, skin inspection techniques, and dressing applications.
- Accessible videos can be included in staff libraries or online platforms.

11. Clinical Guidelines and Toolkits

- Provide access to resources like the **National Pressure Injury Advisory Panel (NPIAP)** guidelines or local protocols.
- Ensure toolkits include checklists, risk assessment tools (e.g., Braden Scale), and care planning templates.

12. Patient and Family Education Materials

- Train nurses on how to use educational pamphlets or videos to teach patients and families about preventing pressure injuries.

13. Mentorship and Peer Education

- Pair less experienced nurses with wound care specialists for on-the-job training.
- Develop a "Pressure Injury Champion" program where trained staff act as resources for their colleagues.

14. Audits and Feedback

- Conduct periodic wound care audits and use feedback sessions as educational moments to address gaps in knowledge or practice.

15. Conferences and Webinars

- Encourage participation in wound care seminars, conferences, and webinars to stay updated on the latest advancements.

Combining these tools ensures a comprehensive, practical, and up-to-date approach to training nurses in preventing and managing pressure injuries.

40. How can parents be involved in the prevention of neonatal pressure injuries?

Parents can play a crucial role in preventing neonatal pressure injuries by being actively involved in their baby's care, collaborating with healthcare professionals, and providing consistent support. Here are some ways they can contribute:

1. Education and Awareness

- Learn about **pressure injuries** (PIs), their causes, and prevention strategies. Healthcare providers can offer guidance on identifying early signs of skin damage and understanding pressure points in neonates.

- Recognize areas at risk, such as the head, heels, and areas under medical devices (e.g., oxygen masks, feeding tubes).

2. Skin Care and Hygiene

- Ensure the baby’s skin is kept clean and dry to minimize skin breakdown. Parents can be instructed on gentle cleansing techniques and the use of appropriate skincare products.
- Moisturize the baby’s skin with safe, approved emollients to prevent dryness, especially in preterm infants.

3. Positioning and Handling

- Assist in **frequent repositioning** to relieve pressure on specific body areas, as advised by healthcare staff. This is especially critical for neonates in intensive care who are unable to move independently.
- Avoid prolonged positioning on bony prominences or under medical devices.
- Encourage "kangaroo care" (skin-to-skin contact), which can reduce pressure on specific areas and improve overall skin health.

4. Monitoring Medical Devices

- Watch for signs of pressure or irritation from medical devices, such as oxygen masks, feeding tubes, or IV lines. Inform healthcare staff immediately if redness, swelling, or indentations are noticed.
- Support healthcare staff in regularly adjusting or repositioning medical devices.

5. Advocating for Preventive Measures

- Ask healthcare teams about the use of **pressure-relieving surfaces** (e.g., specialized mattresses or cushions).
- Ensure barrier creams or protective dressings are used appropriately in high-risk areas.

6. Engagement in Care Plans

- Participate in **care team discussions** and stay informed about interventions and progress.
- Follow instructions on specific techniques or equipment usage to minimize the risk of pressure injuries.

7. Observation and Reporting

- Check the baby’s skin regularly for any early signs of pressure injuries (e.g., redness, blanching, or sores).
- Report any concerns promptly to the healthcare team for timely intervention.

By being educated, vigilant, and proactive, parents can collaborate effectively with healthcare providers to safeguard their neonate’s skin and reduce the risk of pressure injuries.

41. What are the latest scientific findings on the prevention of neonatal pressure injuries?

Neonatal pressure injuries, also known as pressure ulcers, are localized injuries to the skin and underlying tissue resulting from prolonged pressure, often exacerbated by medical devices. Preventing these injuries is crucial due to the delicate nature of neonatal skin and the potential complications that can arise.

Risk Factors and Common Sites: Neonates, especially those in neonatal intensive care units (NICUs), are at heightened risk for pressure injuries due to factors such as prematurity, low birth weight, immobility, and the use of medical devices like endotracheal tubes and nasal continuous positive airway pressure (nCPAP) interfaces. Common sites for these injuries include the occipital region, ears, and areas where medical devices exert pressure.

Dove Press

Preventive Measures:

1. **Risk Assessment:** Regular evaluation using tools tailored for neonates is essential to identify at-risk infants promptly. Implementing structured assessment tools and ongoing education can enhance prevention efforts.

   Frontiers

2. **Medical Device Management:** Ensuring proper placement and fit of devices like nCPAP interfaces is vital. Avoiding excessive pressure and regularly inspecting the skin under these devices can prevent injuries.

   Lippincott Journals

3. **Skin Care:** Maintaining skin integrity through appropriate moisturizing and prompt management of moisture-related issues, such as those from incontinence, is crucial. Using creams containing fatty acids may reduce the incidence of pressure ulcers.

   Wikipedia

4. **Repositioning:** Regularly changing the infant's position helps alleviate prolonged pressure on vulnerable areas. This practice is a cornerstone of pressure injury prevention.

   Wikipedia

5. **Nutritional Support:** Ensuring adequate nutrition supports skin health and overall resilience, though the benefits of specific nutritional interventions require further research.

   Wikipedia

6. **Education and Training:** Ongoing education for healthcare providers about the unique needs of neonates and the importance of pressure injury prevention is essential. Positive attitudes and behaviors among nurses significantly influence prevention practices.

Frontiers

Recent Developments: Advancements in technology, such as AI-driven platforms, are being explored to enhance early identification of patients at risk for pressure injuries, allowing for timely preventive care.

Time

In conclusion, preventing neonatal pressure injuries requires a multifaceted approach that includes regular risk assessments, meticulous device management, diligent skin care, consistent repositioning, adequate nutritional support, and comprehensive education for healthcare providers. Staying informed about the latest research and integrating evidence-based practices are key to safeguarding the health and well-being of neonates.

42. Are there recent studies on the use of innovative materials for preventing or treating pressure injuries?

Recent studies have explored innovative materials and technologies for preventing and treating pressure injuries (PIs). Notable advancements include:

Smart Health Textiles: Research has focused on developing smart clothing designed for individuals with reduced mobility to prevent PIs. These garments incorporate features like thermoregulation, sensing, and antibacterial properties to maintain skin integrity and prevent ulcer formation.

MDPI

Smart Mats for Pressure Monitoring: Innovations such as smart mats equipped with pressure sensors have been developed to monitor body positions and pressure distribution in real-time. These devices alert users and caregivers to prolonged pressure, enabling timely repositioning to prevent PIs.

arXiv

Advanced Wound Dressings: Studies have examined dressings with enhanced properties, such as foam dressings, which provide a moist environment conducive to healing and offer cushioning to redistribute pressure. These dressings are particularly beneficial in treating existing PIs by promoting faster healing and protecting the wound from further trauma.

UEA Eprints

Pressure-Redistributing Support Surfaces: The use of specialized mattresses and overlays, including alternating pressure air mattresses and reactive air surfaces, has been investigated. These support surfaces aim to redistribute pressure and reduce the risk of PI development, especially in high-risk populations.

Welcome

These advancements highlight the ongoing efforts to utilize innovative materials and technologies in the prevention and treatment of pressure injuries, aiming to improve patient outcomes and reduce the incidence of these debilitating conditions.

43. What are the most recognized international guidelines on neonatal pressure injuries?

The most recognized international guidelines addressing neonatal pressure injuries are encompassed within the broader clinical practice guidelines for pressure injury prevention and treatment. Notably, the "Prevention and Treatment of Pressure Ulcers/Injuries: Clinical Practice Guideline," developed collaboratively by the European Pressure Ulcer Advisory Panel (EPUAP), the National Pressure Injury Advisory Panel (NPIAP), and the Pan Pacific Pressure Injury Alliance (PPPIA), offers comprehensive, evidence-based recommendations pertinent to all age groups, including neonates.

International Guideline

The third edition of this guideline was released in November 2019, and work is currently underway for the fourth edition, anticipated for release in 2025.

NPIAP

Additionally, the National Pressure Injury Advisory Panel (NPIAP) has published a white paper titled "Pressure Ulcers in Neonates and Children," which provides insights into the unique considerations for preventing and managing pressure injuries in pediatric populations, including neonates.

Ymaws

These guidelines serve as essential resources for healthcare professionals aiming to implement effective strategies for the prevention and management of pressure injuries in neonatal patients.

44. Are there differences between guidelines in different countries on neonatal pressure injuries?

Neonatal pressure injury guidelines are largely harmonized internationally, thanks to collaborative efforts among major organizations. The European Pressure Ulcer Advisory Panel (EPUAP), the National Pressure Injury Advisory Panel (NPIAP), and the Pan Pacific Pressure Injury Alliance (PPPIA) have jointly developed comprehensive, evidence-based guidelines for the prevention and treatment of pressure injuries, including those in neonates. The most recent edition, released in 2019, is accessible for free online.

International Guideline

These international guidelines are designed to be universally applicable, providing a standardized approach to pressure injury prevention and management across different countries. However, individual nations may adapt these recommendations to align with local healthcare practices, resources, and patient populations. For instance, certain countries might develop specific

protocols or educational materials tailored to their unique clinical settings or cultural considerations.

In Italy, the Italian Society of Wound Care (AISLeC) has translated the Quick Reference Guide of the 2019 International Guideline into Italian, making it more accessible to Italian healthcare professionals.

Epuap

While the core principles of neonatal pressure injury prevention and treatment remain consistent globally, these localized adaptations ensure that guidelines are practical and effective within specific healthcare contexts. Therefore, while there may be minor differences in implementation strategies or emphasis, the foundational guidelines are consistent across countries.

It's also worth noting that work is underway on the fourth edition of the International Pressure Ulcer/Injury Guideline, with an anticipated publication date in early 2025. This upcoming edition aims to incorporate the latest research findings and clinical best practices to further enhance patient care worldwide.

International Guideline

45. How can the quality of the available scientific evidence about neonatal pressure injuries be assessed?

The quality of the available scientific evidence about neonatal pressure injuries (NPIs) can be assessed using a systematic approach, typically incorporating frameworks and tools that evaluate the rigor and reliability of the research. Here are steps and considerations to assess the quality of evidence:

1. Use of Evidence Grading Systems

Several standardized systems are available to assess evidence quality, including:

- **GRADE (Grading of Recommendations, Assessment, Development, and Evaluations):**
  - Evaluates the quality of evidence based on study design, risk of bias, consistency, directness, precision, and publication bias.
  - Assigns evidence quality as high, moderate, low, or very low.
- **SIGN (Scottish Intercollegiate Guidelines Network):**
  - Assesses evidence based on study type and methodological rigor, assigning levels of evidence.
- **CASP (Critical Appraisal Skills Programme):**
  - Provides tools to critically appraise the quality and applicability of individual studies.

2. Evaluate Study Design

- **Randomized Controlled Trials (RCTs):**

    - Considered the gold standard but rare in neonatal populations due to ethical and practical challenges.
- **Observational Studies:**
    - Common in neonatal research. Examine the potential for bias and confounding.
- **Systematic Reviews and Meta-Analyses:**
    - Check if the review used rigorous methods (e.g., PRISMA guidelines) and included high-quality studies.

3. Assess Methodological Quality

- **Risk of Bias:**
    - Were the studies blinded? Was allocation concealed? Were outcome assessments unbiased?
- **Sample Size and Representativeness:**
    - Does the sample size support the conclusions? Does the population reflect the neonatal demographic at risk for pressure injuries?
- **Outcome Measures:**
    - Are the measures clinically relevant, valid, and reliably reported?

4. Analyze Consistency and Precision

- **Consistency:**
    - Do results align across multiple studies? If heterogeneity exists, is it explained?
- **Precision:**
    - Are confidence intervals narrow? Do they demonstrate the strength of the evidence?

5. Assess External Validity (Applicability)

- **Generalizability:**
    - Are the findings applicable to the neonatal population in various clinical settings?
- **Relevance:**
    - Do the interventions, risk factors, and outcomes studied address current neonatal care standards?

6. Look for Comprehensive Reporting

- **Use of Guidelines:**
    - Check whether studies adhered to standards like CONSORT (for RCTs) or STROBE (for observational studies).
- **Transparency:**
    - Are the methods, data, and findings reported transparently to allow replication and validation?

7. Examine Publication Bias

- **Systematic Reviews:**
    - Were unpublished studies or studies with negative results included to reduce bias?
- **Funnel Plots:**

- Used in meta-analyses to detect publication bias.

8. Consider Ethical and Contextual Factors

- Ethical challenges in neonatal research can limit RCTs, leading to reliance on observational data. Acknowledge these limitations when assessing evidence.

9. Engage in Critical Appraisal

Tools like the following can assist in critical appraisal:

- **Joanna Briggs Institute (JBI) Critical Appraisal Tools**
- **Cochrane Risk of Bias Tool**

10. Conduct or Review Systematic Evidence Syntheses

If existing evidence is sparse or of low quality, systematic reviews, meta-analyses, or structured scoping reviews may help synthesize available data and identify gaps.

By applying these principles and tools, the quality of evidence about neonatal pressure injuries can be rigorously and systematically evaluated.

46. What are the legal implications of mismanagement of pressure injuries in neonates?

The legal implications of mismanagement of pressure injuries in neonates can be significant, and they involve several aspects of medical practice, ethics, and law. Some of the key legal considerations include:

1. **Medical Negligence**:
   - If a pressure injury (also known as a pressure ulcer or bedsore) is not identified, treated, or managed appropriately, it could be classified as medical negligence. Medical negligence occurs when a healthcare provider fails to meet the accepted standard of care, leading to harm or injury to the patient. In the case of neonates, their delicate skin and vulnerability require particular attention, and failure to prevent or treat pressure injuries may be seen as a breach of duty.
2. **Standard of Care**:
   - In healthcare, the "standard of care" refers to the level of care that a competent healthcare provider with similar training and experience would provide in similar circumstances. In neonates, especially in those with low birth weight, preterm birth, or other underlying health conditions, the standard of care for preventing and managing pressure injuries is high. Failing to adhere to these standards could result in legal liability.
3. **Informed Consent**:
   - While informed consent typically focuses on adult patients, it is still relevant in the neonatal context, as parents or legal guardians must provide consent for all medical treatments. Mismanagement of pressure injuries could lead to claims of failure to inform the parents of the risks, signs, and necessary preventive measures.

4. **Documentation**:
    - Adequate documentation of the care provided is critical in neonatal care. If a pressure injury develops, proper documentation showing regular assessment, timely interventions, and any preventive strategies used can help defend against allegations of negligence. Failure to document the identification and management of pressure injuries may create a presumption of negligence.
5. **Potential Lawsuits**:
    - Parents or guardians may file lawsuits for damages related to the improper treatment of pressure injuries in neonates. This could include claims for pain and suffering, long-term care costs, and in some cases, punitive damages if the mismanagement was egregious.
6. **Regulatory Consequences**:
    - In cases of severe mismanagement, healthcare providers or institutions may face regulatory consequences from medical boards or licensing agencies. These can include investigations, reprimands, fines, suspension, or even the revocation of medical licenses if it is found that patient safety was compromised due to negligence.
7. **Emotional and Psychological Impact**:
    - The emotional and psychological toll on parents or guardians of a neonate who suffers from a pressure injury can also play a role in legal actions. In some cases, the development of pressure injuries may be seen as an avoidable tragedy that causes significant emotional distress to the family, leading to damages beyond physical injury.

In summary, the legal implications of mismanaging pressure injuries in neonates are multifaceted, including potential claims of medical negligence, breach of standard of care, regulatory scrutiny, and emotional distress for the family. Healthcare providers must take extra care in preventing, identifying, and managing pressure injuries in neonates to avoid these serious legal consequences.

47. How should adverse events related to pressure injuries in neonates be documented?

Adverse events related to pressure injuries in neonates should be documented in a structured, comprehensive, and accurate manner to ensure proper care and prevent recurrence. The documentation should include the following key elements:

1. **Patient Information**:
    - Include the neonate's full name, age, birth weight, and any pertinent medical history, such as gestational age or conditions that predispose the patient to pressure injuries (e.g., prematurity, respiratory support, poor nutrition).
2. **Description of the Pressure Injury**:
    - **Location**: Record the precise anatomical location of the pressure injury.
    - **Stage of Injury**: Use the standardized staging system (e.g., Stage I to IV) to describe the severity of the pressure injury, as per established guidelines.
    - **Size**: Measure the dimensions of the injury (length, width, depth), and document any tunneling or undermining.
    - **Appearance**: Note the color, tissue type (e.g., necrotic, granulation, or slough), and any signs of infection (e.g., purulent discharge, odor).
    - **Pain Level**: Document any pain or discomfort associated with the injury, including neonate's response to touch or interventions.

- **Signs of Infection**: Record the presence of infection such as increased redness, warmth, drainage, or fever.

3. **Contributing Factors**:
   - Identify potential causes or contributing factors such as medical devices (e.g., CPAP masks, tubes), prolonged pressure due to positioning, skin immobility, and impaired circulation.
   - Record whether the injury was related to medical procedures or interventions.
4. **Treatment and Care Provided**:
   - Document the steps taken to address the pressure injury, including pressure-relieving measures (e.g., repositioning, use of special mattresses, or cushions).
   - Include any dressings or topical treatments applied, such as hydrocolloid dressings, antimicrobial treatments, or debridement procedures.
   - If antibiotics or other systemic treatments were administered, note the specific medications, dosages, and treatment duration.
5. **Monitoring and Outcomes**:
   - Document the frequency of monitoring and assessments (e.g., daily or weekly wound checks).
   - Track any changes in the pressure injury's condition (improvement, worsening, or new development).
   - Note if the injury healed, if it required surgical intervention, or if complications (e.g., sepsis) occurred.
6. **Prevention Strategies**:
   - Document preventive measures implemented to avoid further injury, including skin assessments, nutritional support, and proper positioning techniques.
   - Record any educational efforts directed at caregivers or healthcare providers regarding pressure injury prevention.
7. **Interdisciplinary Team Involvement**:
   - Note any consultations or involvement from specialists such as wound care teams, neonatologists, or pediatricians.
   - If the adverse event was reported as part of a clinical incident or quality improvement initiative, ensure it is noted appropriately in the patient's record.

Proper and timely documentation is essential for monitoring the neonate's progress, ensuring the adequacy of interventions, and adhering to healthcare protocols and standards.

48. What are the ethical responsibilities of nurses in preventing pressure injuries?

Nurses have several ethical responsibilities in preventing pressure injuries, which are guided by principles of beneficence (doing good), non-maleficence (avoiding harm), and justice (ensuring fairness and equity). These responsibilities include:

1. **Providing High-Quality Care**: Nurses must use their knowledge, skills, and judgment to prevent pressure injuries by assessing risk factors, implementing preventive strategies, and ensuring appropriate interventions. This aligns with the principle of beneficence, ensuring that patients receive optimal care.
2. **Advocating for Patients**: Nurses are ethically responsible for advocating on behalf of patients to ensure they receive necessary preventive measures, such as regular repositioning, proper nutrition, skin care, and the use of pressure-relieving devices. Advocacy ensures that patient needs are met, especially for vulnerable individuals at higher risk of pressure injuries.

3. **Maintaining Dignity and Comfort**: Nurses must focus on maintaining patient comfort and dignity while preventing pressure injuries. This includes using appropriate techniques for repositioning and providing comfort measures that minimize discomfort or distress during interventions.
4. **Providing Education**: Nurses have an ethical obligation to educate patients, families, and caregivers about the importance of pressure injury prevention, such as the role of mobility, nutrition, and skin care. Education empowers individuals to be active participants in their care.
5. **Ensuring Equity in Care**: Nurses should strive for equity in the prevention of pressure injuries, ensuring that all patients—regardless of age, socioeconomic status, or background—receive equal attention and resources to prevent these injuries.
6. **Reporting and Documentation**: Nurses have an ethical obligation to accurately document risk assessments, interventions, and the condition of the skin. Reporting any signs of potential pressure injuries promptly ensures timely intervention and prevents harm to the patient.
7. **Collaboration with Other Healthcare Professionals**: Ethical responsibility involves collaborating with the entire healthcare team to ensure coordinated care. This can involve working with dietitians, physical therapists, and wound care specialists to address all aspects of pressure injury prevention and care.
8. **Continuous Professional Development**: Nurses must engage in ongoing education and professional development to stay informed about best practices for preventing pressure injuries and maintaining competence in their practice.

By fulfilling these ethical responsibilities, nurses ensure that they are promoting patient well-being, minimizing harm, and delivering care that is both compassionate and professional.

49. How can the balance between skin protection and the clinical management of neonates be achieved?

Achieving a balance between skin protection and the clinical management of neonates requires a multi-faceted approach that takes into account the delicate nature of neonatal skin, the need for effective medical treatments, and the prevention of skin damage. Here are some strategies to ensure this balance:

1. **Gentle Skin Care**: Neonatal skin is thin, fragile, and prone to damage. Use mild, fragrance-free cleansers and moisturizers designed for newborns. Avoid harsh soaps, excessive scrubbing, and products with alcohol or other irritating chemicals.
2. **Hydration**: Neonatal skin is prone to dryness and can become compromised, especially in incubators or under intensive care conditions. Regular moisturizing with appropriate emollients helps maintain skin barrier function, preventing transepidermal water loss.
3. **Temperature and Humidity Control**: Premature and sick neonates have difficulty regulating their body temperature, which can affect their skin. Maintaining a warm and humidified environment (in incubators or under radiant warmers) helps prevent drying and cracking of the skin.
4. **Prevention of Pressure Injuries**: Neonates, especially those in neonatal intensive care units (NICU), may be at risk for pressure injuries due to prolonged positioning. Rotate their position regularly, use soft, breathable bedding, and apply barrier creams to areas at risk for friction or moisture-related injury.
5. **Barrier Creams and Ointments**: When dealing with conditions like diaper dermatitis or the management of central lines, gentle barrier creams (containing zinc oxide or

petroleum jelly) can be used to protect the skin from irritants or excessive moisture. However, they should be applied sparingly to avoid interfering with the absorption of therapeutic agents.

6. **Topical Medications and Treatments**: Topical medications should be applied with caution, considering their potential to affect skin integrity. If topical treatments are needed, use products with low irritation potential and avoid those that can cause skin thinning (e.g., corticosteroids). Ensure that medications such as antifungals or antibiotics are appropriately prescribed, considering the neonatal skin's permeability.
7. **Handling and Dressing**: Minimize the handling of neonates to avoid friction and mechanical damage. When necessary, use soft, breathable clothing, and ensure that the dressing materials (such as bandages or adhesive tapes) are gentle and hypoallergenic. Avoid tight or restrictive clothing that could impair circulation and cause pressure sores.
8. **Skin Surveillance**: Regular monitoring for any signs of skin breakdown, infection, or irritations is essential. In the NICU, this is especially important for neonates with conditions like jaundice, preterm birth, or those requiring intravenous therapies, which can all affect skin health.
9. **Care for Specific Conditions**: Neonates may require specific treatments, such as phototherapy for jaundice. In such cases, ensure appropriate shielding from light to prevent skin damage while balancing the need for treatment. Additionally, appropriate moisturization and monitoring of skin changes are key when neonates are undergoing phototherapy.
10. **Education and Teamwork**: Healthcare professionals should be educated on the best practices for neonatal skin care. This includes training on the application of medications, proper positioning, use of appropriate products, and recognizing the early signs of skin irritation or breakdown.

In summary, achieving a balance requires understanding the unique needs of neonatal skin and providing care that protects the skin while allowing for essential medical treatments. Regular assessment, appropriate interventions, and multidisciplinary collaboration are essential to ensuring the skin remains protected while managing the clinical care of neonates.

50. Are there ethical protocols for research on neonatal pressure injuries?

Yes, there are several ethical protocols and guidelines in place for conducting research on neonatal pressure injuries (also known as pressure ulcers or bedsores) to ensure the safety, dignity, and well-being of neonatal participants. These ethical considerations are particularly crucial due to the vulnerability of neonates and the specific challenges involved in conducting clinical research with this population. The main ethical protocols include:

1. **Informed Consent**:
   - Informed consent is a cornerstone of ethical research. Since neonates are unable to provide consent themselves, it must be obtained from the parents or legal guardians. This consent must be based on clear, comprehensive information about the study's purpose, procedures, risks, and benefits.
   - Special care must be taken to ensure that parents or guardians fully understand the nature of the research, the potential risks involved (including the risk of harm or discomfort to the infant), and the option to withdraw at any time without consequence.
2. **Minimizing Risk and Harm**:

- Neonates are particularly vulnerable, so any research involving them should prioritize minimizing harm. Research protocols should be designed to avoid unnecessary procedures or discomfort. Ethical guidelines emphasize ensuring that any potential benefit to the infant or to broader neonatal care outweighs the risks of the research.
- Pressure injury research should be designed with careful attention to avoiding practices that could exacerbate or cause new pressure injuries or harm the neonate's skin.

3. **Ethical Review and Approval**:
   - Research involving neonates must be approved by an Institutional Review Board (IRB) or ethics committee that is responsible for ensuring that the research complies with ethical standards, including ensuring participant safety and protecting their rights.
   - These committees review research protocols, including risk assessments and the potential benefits of the study to neonatal care.
4. **Confidentiality and Privacy**:
   - Neonatal data should be treated with the highest standards of confidentiality. Personal identifying information must be protected to safeguard the privacy of neonate participants and their families.
   - All data collected must be stored securely, and only authorized personnel should have access.
5. **Equity and Fairness**:
   - Research must ensure that the benefits of the research extend to the populations that are being studied, and that vulnerable populations like neonates are not exploited.
   - Ethical guidelines stress that neonatal studies should avoid unnecessary or unjust recruitment of infants into research, and that there should be equitable access to participation, without bias.
6. **Post-study Care**:
   - If any pressure injuries or other harms occur as a result of the study, neonatal participants should be provided with immediate care and follow-up treatment.
   - Research protocols should include plans for providing any necessary care or compensation if adverse outcomes arise from participation.
7. **Transparency and Integrity**:
   - Research findings should be reported accurately, and researchers should adhere to the highest standards of scientific integrity. Negative or inconclusive results are as valuable as positive ones in guiding clinical practice.
   - All research findings, particularly in the case of neonates, should be shared with the scientific community to improve future care practices.
8. **Beneficence and Non-Maleficence**:
   - Ethical guidelines stress the importance of beneficence (doing good) and non-maleficence (avoiding harm). In neonatal pressure injury research, the potential benefits of the study—such as developing better preventative or treatment strategies for pressure injuries—should outweigh any potential risks.

These ethical protocols are guided by broad principles such as those found in the Declaration of Helsinki and the Belmont Report, which address research ethics in human participants more generally, but they have been specifically tailored to the unique needs and vulnerabilities of neonates in research settings.

51. What technologies are available to monitor the risk of pressure injuries in neonates?

Monitoring the risk of pressure injuries (PIs) in neonates is crucial due to their delicate skin and physiological immaturity. Several technologies and approaches have been developed to assess and prevent pressure injuries in this vulnerable population:

1. **Pressure Mapping Systems**: These systems use sensors embedded in mattresses, bed pads, or blankets to monitor the pressure distribution on a neonate's skin. They can provide real-time data on areas of high pressure, helping healthcare providers reposition the neonate to reduce the risk of developing PIs. Examples include:
   - **Electronic pressure mapping systems** like the **Pediatric Pressure Mapping System** or **Smart Mattresses**.
2. **Skin Temperature Monitoring**: Elevated skin temperature can be an early indicator of pressure injury risk. Technologies like **infrared thermometers** or **continuous skin temperature sensors** can help monitor skin temperature changes, signaling the need for intervention to reduce pressure.
3. **Wearable Sensors**: These sensors can be attached to the neonate's body to monitor changes in pressure, moisture levels, and skin temperature. Some of these devices include:
   - **Smart bandages** or **patch sensors** that provide continuous monitoring of the skin's condition and pressure exposure.
   - **Moisture sensors** integrated into mattresses or clothing to detect excessive moisture, which can increase the risk of PIs.
4. **Telemetry Systems**: Advanced telemetry systems can provide continuous data on pressure points and other factors contributing to PI risk. These systems can send alerts to healthcare staff if critical pressure thresholds are exceeded, prompting immediate repositioning.
5. **Artificial Intelligence (AI) and Machine Learning**: AI-based systems can analyze data from various sources, including pressure maps, temperature sensors, and moisture sensors, to predict the likelihood of pressure injuries. These tools can help healthcare providers identify neonates at high risk and intervene proactively.
6. **Ultrasound Imaging**: Though not widely used for routine monitoring, ultrasound technology can help assess skin and tissue integrity, identifying early signs of damage due to pressure. It can be used in high-risk cases to visualize tissue damage beneath the skin surface.
7. **Automated Repositioning Systems**: Some neonatal care beds are equipped with automated systems designed to adjust the position of the baby periodically, reducing prolonged pressure on any specific area. These systems aim to prevent pressure injuries by ensuring regular movement.

These technologies, combined with clinical monitoring and good nursing practices (such as frequent repositioning, proper skin care, and using appropriate mattresses or supports), can significantly reduce the risk of pressure injuries in neonates.

52. Can the use of skin sensors prevent pressure injuries in neonates?

The use of skin sensors for pressure injury prevention in neonates is a developing area of research. Neonates, particularly those in neonatal intensive care units (NICUs), are at higher risk for pressure injuries due to factors such as fragile skin, immobility, and prolonged medical interventions (e.g., mechanical ventilation, catheters, and positioning aids). Pressure injuries (also

known as pressure ulcers or bedsores) can be a significant concern for neonates in these settings, as their skin is more susceptible to damage.

Skin sensors, often integrated with monitoring systems, can help prevent pressure injuries by continuously tracking the pressure applied to the skin, particularly over bony prominences like the sacrum, heels, and occiput. These sensors can detect areas of high or prolonged pressure and alert healthcare providers, allowing for timely repositioning or other interventions before tissue damage occurs.

Key benefits of using skin sensors in neonates include:

1. **Early Detection**: Sensors can detect changes in skin condition or pressure levels in real-time, allowing for immediate corrective actions.
2. **Reduced Pressure Exposure**: Continuous monitoring can help ensure that pressure is relieved before it leads to tissue ischemia, a major cause of pressure injury development.
3. **Objective Data**: Skin sensors can provide objective, quantifiable data on pressure distribution and duration, which can be used for individualized care plans.
4. **Reduced Manual Checks**: The use of continuous monitoring can reduce the need for frequent manual assessments, allowing caregivers to focus on other aspects of care.

However, some challenges remain in the clinical use of skin sensors for neonates:

- **Sensor Placement**: Accurate placement of the sensors is critical to ensuring effective monitoring. Given the size and fragility of neonatal skin, sensor attachment can be challenging.
- **Cost**: The technology behind skin sensors may be costly, potentially limiting widespread use in some healthcare settings.
- **Comfort**: For neonates, it's crucial that sensors do not cause further discomfort or injury.
- **Long-Term Efficacy**: The long-term benefits and effectiveness of skin sensors in preventing pressure injuries in neonates need more robust clinical evidence and studies.

In conclusion, while skin sensors hold promise as a preventive tool for pressure injuries in neonates, more research is needed to establish their efficacy, optimal usage protocols, and integration into routine neonatal care. They offer a proactive approach to preventing pressure injuries, potentially improving outcomes for vulnerable neonate populations in high-risk settings like NICUs.

53. **Regarding pressure injuries, are there new materials or dressings specifically designed for neonates?**

Yes, there are specialized materials and dressings designed for neonates to prevent and treat pressure injuries. Neonatal skin is particularly delicate and susceptible to damage, necessitating tailored wound care solutions.

Hydrocolloid Dressings: These dressings are flexible and moldable, making them suitable for the unique contours of neonatal skin. They form a gel-like substance upon contact with wound exudate, maintaining a moist environment that promotes healing. Hydrocolloid dressings are effective in protecting the skin from further injury and are recommended for use in neonatal wound care.

Nursing Center

Hydrogel Dressings: Composed primarily of water, hydrogel dressings hydrate the wound bed, aiding in pain relief and facilitating autolytic debridement. They are particularly beneficial for partial-thickness wounds and can be used in neonatal care to support the healing process.

Nursing Center

Foam Dressings: These dressings provide cushioning and absorb exudate, reducing pressure and friction on the skin. They are often used as secondary dressings to protect neonates with conditions like epidermolysis bullosa from movement damage and to prevent the fusion of adjacent rubbing tissues.

Nursing Center

Polymeric Membrane Dressings: These innovative dressings have been shown to reduce tissue edema and inflammation, even in closed injuries like sprains and bruises. They are also the only drug-free dressing shown to reduce pain. Their unique properties make them suitable for the acute management of extravasation injuries in neonates.

WoundSource

It's important to note that while these dressings are beneficial, their use should be guided by clinical judgment and the specific needs of the neonate. Regular assessment and monitoring are essential to ensure optimal healing and prevent complications.

54. What are the benefits of artificial intelligence in managing neonatal pressure injuries?

Artificial intelligence (AI) offers several potential benefits in managing neonatal pressure injuries, especially in the sensitive and complex care environments of neonatal units. Some of these benefits include:

1. **Early Detection and Diagnosis**: AI-based image recognition systems can analyze skin conditions, including pressure injuries, in real-time. They can detect early signs of pressure ulcers by processing images of the skin, often more accurately than the human eye. Early detection allows for timely interventions, reducing the risk of further complications.
2. **Monitoring and Predictive Analytics**: AI can be used to continuously monitor neonatal patients and predict the likelihood of pressure injuries based on data such as patient positioning, skin moisture, and overall health parameters. This predictive approach helps healthcare providers anticipate issues and take preventive measures before the injury occurs.
3. **Personalized Care**: AI can analyze vast amounts of data from electronic health records to identify patterns in a neonate's medical history, risk factors, and previous interventions. This allows for the customization of care plans tailored to the specific needs of each patient, optimizing outcomes.
4. **Decision Support**: AI-driven decision support systems can assist healthcare providers in choosing the most effective interventions for preventing or treating neonatal pressure injuries. By analyzing clinical guidelines, patient data, and best practices, AI can suggest

the most appropriate therapies, including the selection of dressings, positioning techniques, and nutrition strategies.

5. **Reducing Human Error**: In high-stress environments like neonatal intensive care units (NICUs), AI can help minimize human error by providing accurate and timely recommendations. Automated tools for skin assessment and care reminders ensure that the neonate receives optimal attention, reducing the likelihood of overlooked injuries.
6. **Resource Optimization**: AI tools can optimize resource allocation by analyzing patterns in pressure injury development and suggesting targeted interventions. This improves efficiency and helps healthcare teams prioritize resources, especially in resource-limited settings.
7. **Improved Documentation and Reporting**: AI can automate the documentation of neonatal pressure injuries, creating detailed, accurate records of the injury's progress. This provides a clear history of treatment, aids in communication between care teams, and ensures compliance with standards for quality care.
8. **Training and Education**: AI-driven simulations and virtual training tools can educate healthcare professionals on best practices for preventing and managing neonatal pressure injuries. These tools help increase awareness and improve the skills of staff in neonatal care.

Overall, AI enhances the ability to prevent, detect, and treat neonatal pressure injuries in a timely and effective manner, leading to better outcomes and quality of care in neonatal healthcare settings.

55. How are the cost-benefit analyses of advanced technologies in pressure injury prevention evaluated?

The cost-benefit analysis (CBA) of advanced technologies in pressure injury prevention is typically evaluated through a combination of the following steps:

1. Assessment of the Cost of Technology:

- **Initial Investment**: This includes the upfront costs of acquiring advanced technologies such as pressure-relieving mattresses, sensors, or advanced dressings. These costs may vary depending on the type of technology (e.g., high-tech support surfaces, electronic monitoring systems).
- **Maintenance and Operational Costs**: Ongoing costs related to the use and maintenance of these technologies, such as power consumption, maintenance, replacement of components, and consumables (e.g., disposable parts).
- **Training and Personnel**: Costs related to staff training and education required for the proper use of these technologies.

2. Assessment of Benefits:

- **Reduction in Pressure Injuries**: One of the primary benefits is the reduction in incidence or severity of pressure injuries. Studies can estimate the reduced incidence of new pressure ulcers due to the use of advanced technologies.
- **Improved Healing Rates**: If the technology contributes to faster healing of existing pressure injuries, it can reduce the duration and associated costs of wound care and hospitalization.

- **Cost Savings in Long-Term Care**: By preventing pressure injuries, the need for extensive treatments (e.g., debridement, surgery, or long-term wound care) is reduced, leading to cost savings over time. Moreover, preventing pressure injuries can reduce the need for prolonged hospital stays, reducing overall healthcare costs.
- **Quality of Life Improvements**: Although harder to quantify, improvements in patient outcomes, comfort, and quality of life are important benefits. Pressure injuries often lead to pain, discomfort, and functional limitations, so preventing them can significantly improve patient wellbeing.

3. Comparison with Alternatives:

- **Comparison with Standard Care**: The benefits of using advanced technology are compared with the outcomes of standard preventive measures (e.g., regular repositioning, standard foam mattresses).
- **Comparing Technologies**: When more than one advanced technology is available, a comparative analysis of their costs and benefits (such as clinical outcomes, ease of use, and patient preferences) is often conducted.

4. Economic Modeling:

- **Cost-Effectiveness Analysis (CEA)**: This method compares the relative costs and outcomes (e.g., quality-adjusted life years or QALYs) of different interventions. It helps determine if the advanced technology offers better value for money than standard care or other alternatives.
- **Return on Investment (ROI)**: ROI can be used to calculate the financial return of investing in advanced technologies, considering the cost savings from preventing pressure injuries.
- **Cost-Utility Analysis (CUA)**: This involves measuring the benefits in terms of quality-adjusted life years (QALYs), which consider both the quantity and quality of life gained from the technology. This can be particularly useful in healthcare settings, where patient outcomes and quality of life are significant factors.

5. Impact on Healthcare System Efficiency:

- **Hospitalization Costs**: Reducing the frequency of hospital admissions for pressure injuries and related complications can significantly impact overall healthcare costs, including both direct costs (e.g., treatment, medications, staffing) and indirect costs (e.g., lost productivity, long-term disability).
- **Integration with Care Pathways**: Analyzing how the technology fits into current clinical practices and whether it complements or disrupts established care pathways is important in evaluating its overall cost-effectiveness.

6. Long-Term Savings and Sustainability:

- **Preventing Long-Term Complications**: Advanced technologies can prevent not only immediate pressure injuries but also longer-term complications such as infections or sepsis, which can lead to increased healthcare costs.

- **Sustainability**: Considerations of how sustainable the technology is for long-term use in healthcare settings, including issues like ease of scalability across various institutions or care settings, can influence the cost-benefit analysis.

7. Patient-Centered Outcomes:

- **Patient and Family Satisfaction**: Beyond clinical outcomes, the satisfaction of patients and their families with the technology's effectiveness, comfort, and convenience is a valuable factor in the evaluation of advanced technologies.
- **Compliance and Acceptance**: The ease with which patients and caregivers can adopt and maintain the technology (e.g., wearability of devices, non-intrusiveness) is essential for ensuring the technology's success in preventing pressure injuries.

By integrating these aspects into a comprehensive analysis, healthcare providers and policymakers can better assess whether the investment in advanced technologies for pressure injury prevention provides a favorable balance of cost versus benefit, both financially and in terms of patient outcomes.

56. How can pressure injuries affect the psychological well-being of parents?

Pressure injuries, especially in children, can significantly affect the psychological well-being of parents in several ways:

1. **Emotional Stress and Anxiety**: The presence of pressure injuries, which can be painful and slow to heal, often causes emotional distress for parents. Concerns about the child's health and the potential for complications can create ongoing anxiety and worry. The prolonged care and attention required can add to the emotional burden.
2. **Guilt and Helplessness**: Parents may feel guilty about the injury occurring in the first place, particularly if they feel they could have prevented it through better care or monitoring. This can lead to feelings of helplessness, as they may be unable to reverse the injury or relieve the child's pain entirely.
3. **Stress from Increased Care Responsibilities**: Parents often need to manage the increased caregiving demands associated with pressure injuries, including regular wound cleaning, dressing changes, and medical appointments. This added responsibility can create stress and lead to burnout, especially if they are juggling other obligations like work or family care.
4. **Impact on Family Dynamics**: The focus on treating the pressure injury may cause shifts in family routines, potentially straining relationships with siblings or partners. Siblings might feel neglected, while partners may experience tension as both parents cope with the stress in different ways. This can affect the overall family dynamic.
5. **Social Isolation**: Due to the ongoing care needs and the visible nature of the injury, parents may feel socially isolated. They might avoid social situations or feel stigmatized, which can exacerbate feelings of loneliness and further affect their mental health.
6. **Fear of Long-term Impact**: Parents may worry about the long-term consequences of pressure injuries, such as scarring or long-term physical disabilities, which can impact the child's quality of life. These concerns can lead to depression and feelings of despair.
7. **Financial Burden**: The costs associated with wound care supplies, treatments, and possible hospitalizations can place a significant financial burden on families. This financial stress can add another layer of psychological strain.

Overall, parents of children with pressure injuries may experience a range of negative psychological effects, including anxiety, depression, guilt, and stress, which require attention and support to mitigate their impact on the family's mental and emotional well-being.

57. Can parents be involved in the management of pressure injuries?

Yes, parents can play a crucial role in the management of pressure injuries, especially when caring for children or individuals with limited mobility or cognitive impairments. Their involvement can significantly impact both prevention and treatment. Here are some ways parents can participate:

1. **Education and Awareness**: Parents can be educated about pressure injuries, their causes, and preventive strategies. This knowledge helps them monitor at-risk areas and manage early signs of pressure damage.
2. **Positioning and Repositioning**: Parents can assist in regularly repositioning the patient to relieve pressure on vulnerable areas (such as heels, elbows, and sacrum). This is crucial in preventing the development or worsening of pressure injuries.
3. **Skin Care**: Parents can be involved in daily skin checks, ensuring that the skin remains clean, dry, and intact. They can also apply moisturizers or barrier creams to protect the skin.
4. **Nutrition**: Parents can ensure that the patient receives proper nutrition, which is vital for wound healing and skin integrity. Adequate protein, vitamins, and minerals (like vitamin C and zinc) support tissue repair.
5. **Wound Care**: Depending on the severity of the pressure injury, parents may be trained in basic wound care, such as cleaning, dressing, and managing drainage. In some cases, parents can also be taught how to recognize complications, such as infection.
6. **Monitoring for Complications**: Parents can watch for signs of infection (redness, warmth, pus, or fever) or worsening of the pressure injury, enabling prompt medical intervention.
7. **Emotional Support**: Parents can provide emotional and psychological support, helping the patient feel comfortable and less anxious about the treatment process.
8. **Coordination with Healthcare Providers**: Parents can act as a liaison between the healthcare team and the patient, communicating concerns, progress, or changes in the patient's condition.

In summary, with proper training and guidance from healthcare professionals, parents can be an integral part of managing pressure injuries, helping ensure effective prevention and care.

58. What communication strategies are useful for explaining pressure injuries to parents?

When explaining pressure injuries (also known as pressure ulcers or bedsores) to parents, it's important to use clear, compassionate, and educational communication strategies to ensure they understand both the condition and its management. Here are some strategies that can be effective:

1. Use Simple, Clear Language

- Avoid medical jargon, and explain the condition in a way that parents can easily understand.
- For example, explain that pressure injuries occur when prolonged pressure on the skin reduces blood flow, leading to tissue damage.

2. Use Visual Aids

- Show pictures or diagrams of pressure injuries in different stages to give a clear understanding of what the injury looks like.
- Visual aids can help parents understand the seriousness of the condition and how it develops.

3. Empathy and Active Listening

- Show empathy for the emotional and physical stress parents may be experiencing. Acknowledge their concerns and fears.
- Use open-ended questions like "What questions do you have?" or "How do you feel about what we've discussed so far?"

4. Explain Prevention Measures

- Emphasize the importance of prevention by explaining strategies such as regular repositioning, maintaining skin hygiene, and ensuring proper nutrition.
- Provide practical advice on how parents can prevent further injury, such as changing the position of a patient every 2 hours or using specialized mattresses or cushions.

5. Use Analogies

- Analogies can make complex medical conditions more relatable. For example, compare pressure injuries to a garden plant that gets wilted and damages when it doesn't get enough water or sunlight (blood flow).

6. Address Emotional Concerns

- Reassure parents that with proper care, pressure injuries can be treated and prevented in the future. Discuss any treatment plans clearly, and make them feel involved in the process.
- Address any feelings of guilt or concern that parents may have by emphasizing that pressure injuries are common, particularly in individuals who are immobile or have limited sensation.

7. Set Realistic Expectations

- Discuss the healing process, which may take time, and set realistic expectations about what is needed for recovery.

- Help parents understand the ongoing care and monitoring that may be necessary to ensure proper healing.

8. Offer Resources

- Provide written materials or resources such as brochures, websites, or videos that offer more detailed information on pressure injury care and prevention.
- Encourage parents to ask for help or clarification whenever they need it.

9. Collaborate and Empower

- Involve parents in the decision-making process regarding care, as empowering them helps reduce anxiety and gives them a sense of control.
- Offer hands-on demonstrations if needed, showing how to perform tasks such as repositioning, dressing changes, or skin inspections.

10. Follow-up Support

- Offer to follow up with parents regularly to answer any ongoing questions or concerns and ensure they feel supported throughout the care process.

By using these strategies, you can help parents feel more confident in managing and preventing pressure injuries while ensuring that their concerns are addressed.

59. Do pressure injuries affect the bond between parents and neonates?

Pressure injuries (also known as bedsores or pressure ulcers) can have an impact on the bond between parents and neonates, although the direct influence may depend on various factors, including the severity of the injury, the circumstances surrounding the birth, and the healthcare setting.

Here are a few potential ways pressure injuries may affect the bond between parents and neonates:

1. **Emotional Stress and Anxiety**: The presence of a pressure injury in a neonate may lead to emotional distress for the parents. Concern over the baby's well-being, discomfort, and the possible long-term implications of pressure injuries might lead to heightened anxiety and worry. This emotional strain could make it harder for parents to focus on bonding activities, such as holding and nurturing the baby.
2. **Interruption of Parent-Infant Interaction**: Severe pressure injuries may require medical interventions that could interfere with parent-infant interaction. The need for specialized care, such as dressing changes or positioning adjustments to avoid further injury, may limit the amount of time parents can spend physically close to their infant, potentially reducing bonding opportunities like skin-to-skin contact.
3. **Perception of Caregiving Ability**: Parents may feel guilt or a perceived failure in their ability to protect their infant from harm, particularly if the pressure injury develops due to prolonged hospitalization, preterm birth, or medical procedures. This feeling could

influence their confidence and emotional connection with their baby, which may affect bonding.

4. **Medical Interventions and Separation**: If the pressure injury is serious enough to require hospitalization in a neonatal intensive care unit (NICU), the neonate may be separated from the parents for extended periods, potentially hindering bonding. Furthermore, medical interventions and treatment regimens may require strict routines that prevent the parents from providing the comforting presence they desire.
5. **Impact on Long-Term Development**: In severe cases where pressure injuries lead to complications or long-term health issues, parents might experience stress as they cope with their child's recovery or ongoing care. This concern could shift the focus away from emotional bonding and towards the infant's medical needs, further complicating the bonding process.

However, it's important to note that pressure injuries in neonates are typically preventable with proper care and early intervention. Hospitals and neonatal care units often have protocols in place to minimize the risk of these injuries. If the parents are educated and involved in their infant's care, they may feel more empowered and connected to their baby, which can help strengthen the bond despite the challenges posed by medical conditions like pressure injuries.

60. How can healthcare staff be sensitized to the importance of preventing pressure injuries?

Sensitizing healthcare staff to the importance of preventing pressure injuries (also known as pressure ulcers or bedsores) requires a multi-faceted approach that fosters awareness, understanding, and action. Here are some key strategies:

1. **Education and Training:**
   - **Regular Training Programs**: Provide staff with ongoing education about the causes, risk factors, and stages of pressure injuries. Training should include up-to-date guidelines on prevention, such as the use of risk assessment tools (e.g., Braden Scale) and best practices for patient care.
   - **Workshops and Seminars**: Conduct workshops to address specific challenges related to preventing pressure injuries in various settings, including ICU, surgical wards, and long-term care facilities.
   - **Interactive Learning**: Utilize case studies, simulations, or role-playing to help staff recognize high-risk patients and develop the skills to prevent and manage pressure injuries effectively.
2. **Leadership Support and Commitment:**
   - **Management Buy-In**: Leadership should actively support pressure injury prevention efforts. This includes allocating resources for prevention measures and setting clear, achievable goals for reducing incidence rates.
   - **Policy and Protocol Development**: Ensure that there are clear, evidence-based guidelines and policies for pressure injury prevention. Staff should be trained to implement these protocols consistently in their daily practice.
3. **Multidisciplinary Collaboration:**
   - **Team Approach**: Foster a collaborative approach that involves nursing staff, physicians, physical therapists, dietitians, and other healthcare professionals to address all factors that contribute to pressure injury prevention.
   - **Regular Team Meetings**: Hold regular discussions on pressure injury prevention during team meetings to ensure everyone is aligned on strategies and challenges.

4. **Use of Risk Assessment Tools:**
    - **Early Identification of High-Risk Patients**: Encourage the use of validated risk assessment tools, such as the Braden Scale, to assess patients upon admission and regularly during their hospital stay.
    - **Individualized Care Plans**: Use risk assessment results to create individualized care plans that address each patient's specific needs, including mobility, nutrition, and skin care.
5. **Practical Interventions:**
    - **Frequent Repositioning**: Reinforce the importance of regularly repositioning patients, especially those with limited mobility, to reduce pressure on vulnerable areas.
    - **Use of Specialized Equipment**: Ensure that staff is aware of and trained in using pressure-relieving devices, such as specialized mattresses, cushions, and offloading devices.
6. **Patient and Family Involvement:**
    - **Education for Patients and Families**: Teach patients and their families about the risks of pressure injuries and the importance of maintaining mobility and repositioning, especially for patients in long-term care.
    - **Promote Active Involvement**: Encourage families to participate in the prevention process by ensuring that the patient's needs are met, including repositioning and skin inspection.
7. **Data Collection and Feedback:**
    - **Regular Audits and Monitoring**: Monitor and track pressure injury rates within the healthcare facility to identify trends and areas for improvement.
    - **Provide Feedback**: Share the results of audits and assessments with the team to highlight successes and areas needing attention. Recognize staff efforts in preventing pressure injuries, and use data to motivate improvements.
8. **Cultural Change:**
    - **Foster a Prevention-Oriented Culture**: Make pressure injury prevention part of the institution's culture. This means encouraging staff to view prevention as a routine part of care, not just an additional task.
    - **Encourage Reporting**: Create a non-punitive environment where staff feel comfortable reporting potential issues or incidents related to pressure injuries without fear of blame.

By combining education, leadership support, teamwork, and practical interventions, healthcare staff can become more sensitized to the importance of preventing pressure injuries, ultimately improving patient care and outcomes.
